# A molecular map of lung neuroendocrine neoplasms

**DOI:** 10.1093/gigascience/giaa112

**Published:** 2020-10-30

**Authors:** Aurélie A G Gabriel, Emilie Mathian, Lise Mangiante, Catherine Voegele, Vincent Cahais, Akram Ghantous, James D McKay, Nicolas Alcala, Lynnette Fernandez-Cuesta, Matthieu Foll

**Affiliations:** Section of Genetics, International Agency for Research on Cancer (IARC-WHO), 150 cours Albert Thomas, 69372 Lyon CEDEX 08, France; Section of Genetics, International Agency for Research on Cancer (IARC-WHO), 150 cours Albert Thomas, 69372 Lyon CEDEX 08, France; Section of Genetics, International Agency for Research on Cancer (IARC-WHO), 150 cours Albert Thomas, 69372 Lyon CEDEX 08, France; Section of Genetics, International Agency for Research on Cancer (IARC-WHO), 150 cours Albert Thomas, 69372 Lyon CEDEX 08, France; Section of Mechanisms of Carcinogenesis, International Agency for Research on Cancer (IARC-WHO), 150 cours Albert Thomas, 69372 Lyon CEDEX 08, France; Section of Mechanisms of Carcinogenesis, International Agency for Research on Cancer (IARC-WHO), 150 cours Albert Thomas, 69372 Lyon CEDEX 08, France; Section of Genetics, International Agency for Research on Cancer (IARC-WHO), 150 cours Albert Thomas, 69372 Lyon CEDEX 08, France; Section of Genetics, International Agency for Research on Cancer (IARC-WHO), 150 cours Albert Thomas, 69372 Lyon CEDEX 08, France; Section of Genetics, International Agency for Research on Cancer (IARC-WHO), 150 cours Albert Thomas, 69372 Lyon CEDEX 08, France; Section of Genetics, International Agency for Research on Cancer (IARC-WHO), 150 cours Albert Thomas, 69372 Lyon CEDEX 08, France

**Keywords:** carcinoids, lung cancer, neuroendocrine neoplasms, rare cancers, genomics, Tumormap, lungNENomics project

## Abstract

**Background:**

Lung neuroendocrine neoplasms (LNENs) are rare solid cancers, with most genomic studies including a limited number of samples. Recently, generating the first multi-omic dataset for atypical pulmonary carcinoids and the first methylation dataset for large-cell neuroendocrine carcinomas led us to the discovery of clinically relevant molecular groups, as well as a new entity of pulmonary carcinoids (supra-carcinoids).

**Results:**

To promote the integration of LNENs molecular data, we provide here detailed information on data generation and quality control for whole-genome/exome sequencing, RNA sequencing, and EPIC 850K methylation arrays for a total of 84 patients with LNENs. We integrate the transcriptomic data with other previously published data and generate the first comprehensive molecular map of LNENs using the Uniform Manifold Approximation and Projection (UMAP) dimension reduction technique. We show that this map captures the main biological findings of previous studies and can be used as reference to integrate datasets for which RNA sequencing is available. The generated map can be interactively explored and interrogated on the UCSC TumorMap portal (https://tumormap.ucsc.edu/?p=RCG_lungNENomics/LNEN). The data, source code, and compute environments used to generate and evaluate the map as well as the raw data are available, respectively, in a Nextjournal interactive notebook (https://nextjournal.com/rarecancersgenomics/a-molecular-map-of-lung-neuroendocrine-neoplasms/) and at the EMBL-EBI European Genome-phenome Archive and Gene Expression Omnibus data repositories.

**Conclusions:**

We provide data and all resources needed to integrate them with future LNENs transcriptomic studies, allowing meaningful conclusions to be drawn that will eventually lead to a better understanding of this rare understudied disease.

## Background

Lung neuroendocrine neoplasms (LNENs) are rare understudied diseases with limited therapeutic opportunities. LNENs include poorly differentiated and highly aggressive lung neuroendocrine carcinomas (NECs)—i.e., small-cell lung cancer (SCLC) and large-cell neuroendocrine carcinoma (LCNEC)—as well as well-differentiated and less aggressive lung neuroendocrine tumors (NETs), i.e., typical and atypical carcinoids (WHO classification 2015 [1]). Over the past years several genomic studies have investigated the molecular characteristics of these diseases to provide some evidence for more personalized clinical management [2–8]. Although lung NECs and NETs are broadly considered different diseases, several recent studies have suggested that they may share some molecular characteristics [7,9–12]. However, owing to the rarity of these diseases, the sample sizes of these studies individually are limited, and the integration of independent datasets is not an easy task.

Providing a way to interactively visualize and analyze these pan-LNEN data would be of great interest for the scientific community, not only to further explore the proposed molecular link between lung NECs and NETs but also to integrate data from studies including fewer samples to reach the statistical power needed to draw meaningful conclusions.

## Data Description

Recently [7], we performed the first integrative and comparative genomic analysis of LNEN samples from all histological types, based on newly sequenced data: whole-exome sequencing (WES) data (16 samples), whole-genome sequencing (WGS) data (3 samples), RNA-sequencing (RNA-Seq) data (20 samples), and EPIC 850K methylation data (76 samples), as well as publicly available data. These data correspond to the most extensive multi-omic dataset of LNENs, including the first methylation data for LCNEC and the first molecular characterization of the rarest LNEN subtype (atypical carcinoids) [7]. This dataset, which provides the missing pieces for a complete molecular characterization of LNENs, has been deposited at the EMBL-EBI European Genome-phenome Archive (EGA accession No. EGAS00001003699). To facilitate the reuse of the data generated for the previous publication [7], we provide here a complementary data descriptor by outlining the pre-processing and quality control (QC) steps performed on each omic dataset available on EGA.

Also, other studies have generated sequencing data and performed a molecular characterization of LNEN samples: pulmonary carcinoids (mostly typical carinoids) have been characterized by Fernandez-Cuesta et al. [4] and Laddha et al. [8], LCNEC by George et al. [6], and SCLC by George et al. [5] and Peifer et al. [2]. We therefore generate the first pan-LNEN molecular tumor map by integrating the transcriptomic data from Alcala et al. [7] and the other published LNEN transcriptomic data [2,4–6, 8]. This map provides an interactive way to explore the molecular data and allows statistical interrogation, based on the UCSC TumorMap portal [13]. The integrated transcriptomic dataset resulting from these studies is available on GitHub [14].

### Data quality controls

**Figure 1:**
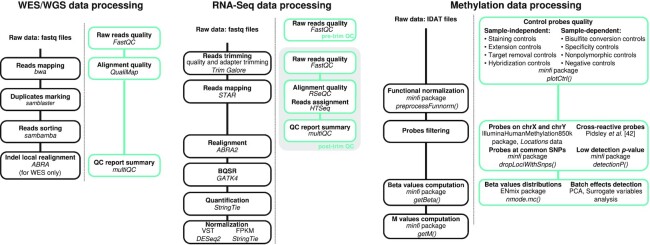
Bioinformatics workflows for data processing and associated quality controls (QC; green boxes). Bioinformatics tools used for the processing of the WES/WGS data, RNA-Seq, and methylation data are represented in the left, middle, and right panels, respectively.

Fig. 1 provides a schematic view of the pre-processing steps and the associated QC performed for each omic dataset generated by Alcala and colleagues [7]. An overview of the available omics and clinical data for each sample is provided in Supplementary Table 1.

**Table 1:** General statistics associated with the quality controls of the WES and WGS data

Sample	Sequencing	Median coverage	Total No. reads (M)	>30× (%)	Aligned (%)	GC content (%)	Median insert size	Duplicates (%)
LNEN002	WES	148	113.3	95.5	99.7	53.7	194	13.9
LNEN003	WES	146	110.3	95.8	99.7	53.7	194	13.4
LNEN004	WES	150	115.3	95.4	99.8	54.3	193	13.1
LNEN005	WES	135	103.4	94.7	99.8	54.0	195	12.1
LNEN006	WES	126	93.6	94.6	99.8	53.5	197	12.5
LNEN007	WES	145	116.3	94.4	99.8	54.5	195	14.8
LNEN009	WES	123	98.4	92.9	99.7	54.1	195	12.4
LNEN010	WES	138	104.1	95.0	99.7	53.3	196	13.4
LNEN011	WES	161	125.8	95.8	99.8	54.3	196	14.8
LNEN013	WES	131	99.2	94.3	99.8	53.5	193	13.0
LNEN014	WES	132	102.6	94.0	99.8	54.1	195	13.3
LNEN015	WES	148	111.3	95.7	99.6	54.1	197	10.1
LNEN016	WES	133	98.0	94.3	99.6	54.3	194	9.0
LNEN017	WES	158	116.4	95.9	99.6	54.1	192	8.9
LNEN020	WES	187	144.7	96.6	99.7	53.6	192	14.5
S00716_B	WES	133	99.8	95.4	99.7	52.8	194	14.3
LNEN041	WGS	36	923.5	77.5	98.9	41.0	366	13.3
LNEN042	WGS	41	993.7	88.1	98.8	41.5	388	9.4
LNEN043	WGS	43	1033.1	89.7	99.3	41.6	392	8.8

GC: guanine-cytosine.

### WES and WGS data

WES and WGS were performed, respectively, on 16 and 3 fresh-frozen atypical carcinoids in the Cologne Centre for Genomics and the Centre National de Recherche en Génomique Humaine. For WES, the SeqCap EZ v2 Library capture kit from NimbleGen (44 Mb) and the Illumina HiSeq 2000 machine (Illumina Inc., San Diego, CA, USA) were used for the sequencing. For WGS, the Illumina TruSeq DNA PCR-Free Library Preparation Kit was used for library preparation and the HiSeqX5 platform from Illumina for the sequencing as described in [7]. The sequencing reads from the 16 atypical carcinoids' whole exomes and the 3 carcinoids' whole genomes were processed using the in-house Nextflow [15] workflow available at the IARCbioinfo/alignment-nf [16] GitHub repository, revision No. 9092214665. The pipeline consists in 3 steps: mapping reads to the reference genome (GRCh37), marking duplicates, and sorting reads using bwa v0.7.12-r1044 (BWA, RRID:SCR_010910) [17], samblaster v0.1.22 (samblaster, RRID:SCR_000468) [18], and sambamba v0.5.9 [19], respectively. For WES samples, local realignment using ABRA v0.97b (ABRA, RRID:SCR_003277) [20] was then run.

The QCs of the WES and WGS data were performed using FastQC v0.11.8 (FastQC, RRID:SCR_014583) [21] and QualiMap v2.2.1 (QualiMap, RRID:SCR_001209) [22] using the in-house Nextflow [15] workflows available at IARCbioinfo/fastqc-nf [23] and IARCbioinfo/qualimap-nf [24] repositories, respectively, and the results aggregated using MultiQC v1.7 (MultiQC, RRID:SCR_014982) [25] (Fig. 1, left panel).

**Figure 2:**
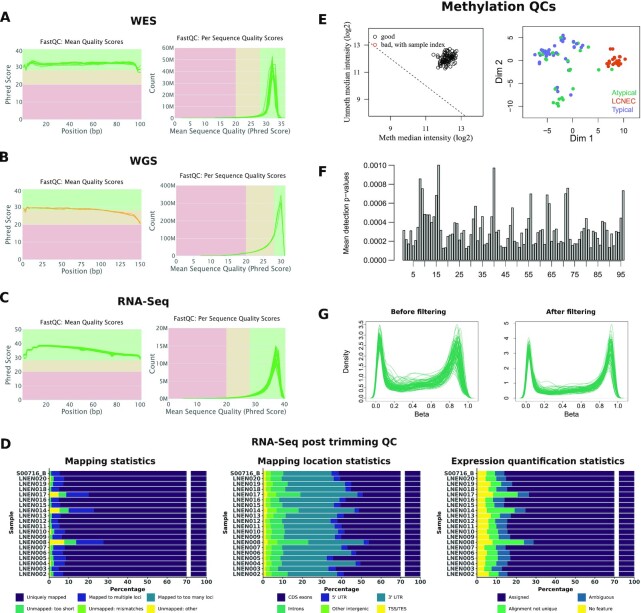
Quality control (QC) performed on each omic dataset. **(A)** Read QC using FastQC for WES data. **(B)** Read QC using FastQC for WGS data. **(C)** Read QC using FastQC for RNA-Seq data. For A, B, and C, the left panels correspond to the sequence quality plots, the x-axis representing the base position in the read and the y-axis the mean quality value; the right panels correspond to the per sequence quality score plots, the x-axis representing the mean quality score and the y-axis the number of reads. **(D)** QC of the RNA-Seq data after trimming. *Left:* Bar plot representing the percentage of reads uniquely mapped (“Uniquely mapped”), mapped to multiple loci (“Mapped to multiple loci” or “Mapped to too many loci” if the number of loci is >10), unmapped because the mapped reads’ proportion was too small (“Unmapped: too short”), unmapped because of too many mismatches (“Unmapped: mismatches”), or unmapped for other reasons (“Unmapped: other”). *Middle:* Cumulative bar plot representing the percentages of reads mapped, using RSeQC, at different locations in the genome (exons, introns, 5′ and 3′ untranslated transcribed region [UTR], intergenic regions, TSS, and TES). *Right:* Cumulative bar plot representing the cumulative percentages associated with the different read assignments using HTSeq (“Assigned”: reads assigned to 1 gene, “Ambiguous”: reads assigned to multiple overlapping genes, “Aligned not unique”: reads assigned to multiple non-overlapping genes, “No Feature”: reads assigned to none of the features). **(E)**  *Left:* Samples’ quality based on log median intensities. The x-axis and y-axis correspond to the median of log_2_ methylated and unmethylated intensities, respectively. *Right:* Representation of the between-sample similarities based on the 2 first multidimensional scaling dimensions. **(F)** Histogram of the median detection *P*-value for each sample. **(G)** Distribution of the β-values for each sample before and after the filtering step (left and right panel, respectively).

Fig. 2A and B show the per base sequence quality scores (left panels) and the per sequence mean quality scores (right panels). Regarding the per base sequence quality scores, the majority of the base calls were of very good quality (>28, green area, Fig. 2A left panel) and of reasonable quality (>20, orange area, Fig. 2B left panel) for WES and WGS data, respectively. The most frequently observed sequence mean quality score was ∼30 for both techniques, which is equivalent to an error probability of 0.1%. Table 1 provides the general statistics associated with the WES and WGS QCs. The observed median coverage for each sample was above the expected coverage (30× for the WGS samples and 120× for the WES samples). Concerning the alignment quality, all WES samples had >99% of the reads aligned and all WGS samples had >98% of the reads aligned.

### RNA-Seq data

RNA-Seq was performed on 20 fresh-frozen atypical samples. The Illumina TruSeq RNA sample preparation Kit was used for library preparation and the Illumina TruSeq PE Cluster Kit v3 and the Illumina TruSeq SBS Kit v3-HS kits were used on an Illumina HiSeq 2000 sequencer. The data generated were processed in 5 steps (Fig. 1, middle panel): (i) read trimming using Trim Galore v0.6.5 (Trim Galore, RRID:SCR_011847) [26], (ii) read mapping to the reference genome (GRCh38, gencode version 33 from bundle CTAT from 6 April 2020 [27]) using STAR v.2.7.3a (STAR, RRID:SCR_015899) [28], (iii) realignment of the reads using ABRA2 v2.22 (ABRA, RRID:SCR_003277) [29], (iv) base quality score recalibration using GATK4 v4.0.5.1 (GATK, RRID:SCR_001876) [30, 31], and (v) gene expression quantification using StringTie v2.1.1 (StringTie, RRID:SCR_016323) [32]. FastQC v.0.11.9 (FastQC, RRID:SCR_014583) [21], RSeQC v3.0.1 (RSeQC, RRID:SCR_005275) [33], and HTSeq v0.12.4 (HTSeq, RRID:SCR_005514) [34] were used to control the raw read quality and assignments, and the results aggregated using MultiQC v1.7 (MultiQC, RRID:SCR_014982) [25]. These steps were performed using our in-house Nextflow [15] pipelines available at the following GitHub repositories: IARCbioinfo/RNAseq-nf [35] release v2.3, IARCbioinfo/abra-nf [36] release v3.0, IARCbioinfo/BQSR-nf [37] release v1.1, and IARCbioinfo/RNAseq-transcript-nf [38] release v2.1.

Fig. 2C shows that the base calls, before trimming, are of good quality because all samples have a mean per base sequence quality score >28 (left panel) and for all samples the most frequently observed per sequence mean quality is >35, corresponding to an error probability of 0.03% (right panel). None of the samples presented >1% of over-represented sequences, which ensures a proper library diversity. RSeQC was used to control the alignment quality and to assign mapped reads to different genomic features (coding regions, introns, intergenic regions, TSS, TES). Fig. 2D (left panel) shows that every sample had >70% of reads uniquely mapped and the read distribution for each sample is represented in Fig. 2D (middle panel). All samples had >75% reads mapped in coding regions (CDS-exons, 5′ and 3′ untranslated transcribed region exons). The read counting was performed at the gene level for 59,607 genes (genecode annotation, release 33) using HTSeq [34]. Fig. 2D (right panel) shows the read assignments; the percentage of assigned reads ranges from 71.3 to 87.3%. STAR, RSeQC, and HTSeq metrics for each sample are provided in Supplementary Tables 2–4. Note that 3 samples, LNEN008, LNEN014, and LNEN017, have a higher proportion of reads classified as “Unmapped too short” and “Mapped to multiple loci” (Fig. 2D, left panel), reads mapped in intronic regions (Fig. 2D, middle panel), and a lower proportion of reads assigned by HTSeq (Fig. 2D, right panel) in comparison with the other samples. Unexpected results concerning those samples should thus be considered with caution.

Finally, to apply dimensionality reduction methods to the RNA-Seq data (see below), the DESeq2 package v1.26.0 (DESeq2, RRID:SCR_015687) [39] was used to transform the read counts obtained using StringTie to variance-stabilized read counts (vst), enabling the comparison of samples with different library sizes. To reduce sex influence on expression profiles, the genes located on sex chromosomes were not considered for subsequent analyses. Genes located on the mitochondrial chromosome were also not considered.

### Methylation data

The methylation analyses were performed on the basis of the EPIC 850K methylation arrays and the Infinium EPIC DNA methylation beadchip platform (Illumina) for 33 typical carcinoids, 23 atypical carcinoids, 20 LCNECs, and 19 technical replicates in total. These arrays interrogate >850,000 CpGs and contain internal control probes that can be used to assess the overall efficiency of the sample preparation steps. The raw intensity data (IDAT files) were processed using the R package minfi v.1.24.0 (minfi, RRID:SCR_012830) [40]. Fig. 1 (right panel) provides the packages, functions, and publication used for the data processing, QC, and filtering steps as implemented in the IARCbioinfo/Methylation_analysis_scripts [41] GitHub repository.

Fig. 2E shows that no outliers were detected: (i) the left panel, representing the median log_2_ of the methylated and unmethylated intensities, indicates that all samples cluster together with a log median intensity >11 for both channels, which supports the absence of failed samples; (ii)in the right panel, the multidimensional scaling plot shows that the samples cluster together by histological groups. We used the depectionP function (minfi package), which compares the DNA signal to the background signal based on the negative control probes to provide a detection *P-*value per probe, lower *P-*value indicating reliable CpGs. Fig. 2F represents the mean detection *P-*values per sample and shows that all samples' mean detection *P-*values were <0.01. To correct for the variability identified in the control probes, a normalization step was applied to the raw intensities using the preprocessFunnorm function from minfi.

After between-array normalization, different sets of probes that could generate artifacts were removed successively from the methylation dataset: (i) 19,634 probes on the sex chromosomes, in order to identify differences related to tumors but unrelated to sex chromosomes; (ii) 41,818 cross-reactive probes, which are probes co-hybridizing with multiple CpGs on the genome and not only to the one for which it has been designed [42]; (iii) 10,588 probes associated with common SNPs (present in dbSNP build 137); (iv) 24,363 probes with multi-modal β-value distribution; and (v) 9,697 probes having a detection *P*-value >0.01 in ≥1 sample. Supplementary Table 5 lists the sets of filtered probes. To assess the experimental quality of the assay, the distributions of the β-values were analyzed. As described previously, probes with multi-modal distributions were removed at the filtering step and overall distributions of β-values for each sample before and after filtering were plotted (Fig. 2G). As expected, after filtering all samples showed a bimodal profile, indicative of the good quality of the experiment. No experimental batch effects were identified after functional normalization (see Supplementary Fig. 33 from [7]). Based on all the QCs performed, none of the samples analyzed were identified as outlier. However, 1 sample available on EGA (201414140007_R06C01) was removed from the analyses because it came from a metastatic tumor rather than the primary tumor. Sample metadata are provided in Supplementary Table 6.

## Generation of an integrative molecular map

Here we have generated a pan-LNEN molecular map with the whole-transcriptomic (RNA-Seq) data available from individual studies of each LNEN tumor type [2,4–8]. This dataset includes the RNA-Seq data for a total of 51 SCLCs, 69 LCNECs, and 118 carcinoids including 40 atypical and 75 typical carcinoids. The different data underwent the same processing steps described above because the generation of the molecular map requires a homogenized dataset.

### Dimensionality reduction using UMAP

#### UMAP method

The pan-LNEN map was obtained using the Uniform Manifold Approximation and Projection (UMAP) method [43] on the genes with the most variable expression (genes explaining 50% of the total variance). UMAP is a dimensionality reduction method based on manifold learning techniques, which are adapted to non-linear data in contrast with the commonly used principal component analysis (PCA) method. First, it builds a topological representation of the high-dimensional data, and second it finds the best low-dimensional representation of this topological structure [43]. UMAP representations were generated using the umap function from the R package umap (v. 0.2.5.0) [44]. All the parameters were set to their default values except the n_neighbors parameter. This parameter defines the number of neighbors considered to learn the structure of the topological space. Varying this parameter from small to large values enables the user to find a trade-off between local and global preservation of the space, respectively. To preserve the global structure of the data (see “quality control of the UMAP projection” section below), we built the pan-LNEN map setting the n_neighbors parameter to 238, which corresponds to the total number of samples.

#### Biological interpretation of the pan-LNEN TumorMap

**Figure 3:**
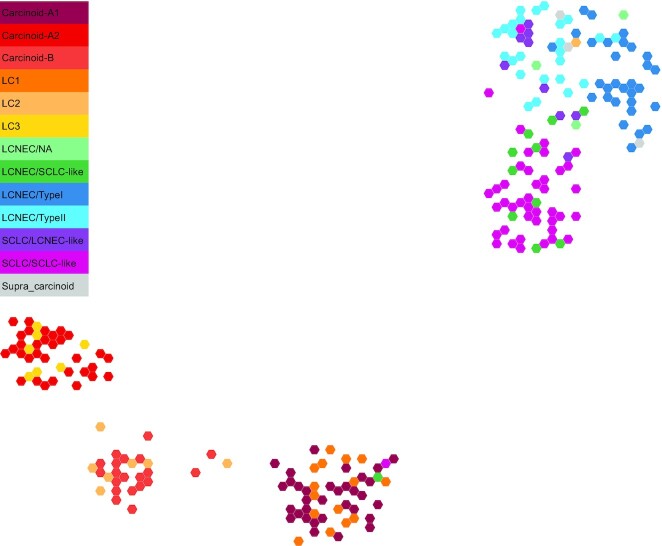
Two-dimensional projection of LNEN transcriptome data using UMAP. The representation was obtained from the TumorMap portal, using the hexagonal grid view, each hexagonal point representing a LNEN sample. Point colors correspond to the molecular clusters defined in the previous publications.

Fig. 3 shows the pan-LNEN map available on TumorMap [45] (see “Reuse potential” section below), with colors representing the main molecular subtypes. To evaluate the accuracy of the generated pan-LNEN map we first verified whether it was consistent with the main biological findings from the original studies, in particular whether it represented the molecular subtypes of LNENs previously identified, and their relationship with histological types. We specifically tested whether groups of samples previously described as having discordant molecular and histopathological features were identified in our map. To do so, given a focal molecular subtype and 2 reference histopathological types, we assessed whether samples from the focal molecular subtype were closer to 1 of the 2 references using a 1-sided Wilcoxon test between the Euclidean distances of samples to the centroid of each reference type.

First, the SCLC/LCNEC-like samples [6], which are histological SCLCs presenting the molecular profile of LCNEC, tend to cluster with the LCNECs rather than with the SCLCs (Wilcoxon *P* = 6.2 × 10^−4^). Similarly, the LCNEC/SCLC-like samples [6], which are histological LCNECs having the molecular profile of SCLC, tend to cluster with the SCLCs rather than with the LCNECs (Wilcoxon *P* = 3.3 × 10^−3^). In 2018, George et al. showed also that LCNEC samples can be subdivided into Type I and Type II molecular groups [6]. We observed that the Type I and Type II LCNECs were closer to each other than to the SCLC/SCLC-like (Wilcoxon *P* = 9.9 × 10^−14^) and that SCLC/LCNEC-like samples were closer to Type II than to Type I LCNECs [6] (Wilcoxon *P* = 3.9 × 10^−3^). Like the LCNECs, pulmonary carcinoids have been subdivided into molecular groups. Alcala et al. [7] identified 3 clinically relevant molecular clusters, using a multi-omics factor analysis: Carcinoid A1, Carcinoid A2, and Carcinoid B [7]. In the pan-LNEN map generated using UMAP, those 3 clusters are clearly visible (Fig. 3) and, respectively, correspond to the 3 clusters identified in [8] named LC1, LC3, and LC2. Also, in the study from Alcala and colleagues [7], 2 carcinoids that clustered with the carcinoids B (S00118 and S00089) were borderline and located between cluster A1 and B. Similarly, an LCNEC sample and an SCLC sample clustered with the carcinoids A1 [7]. These observations are also visible on the TumorMap representation. Finally, in the same study, a novel entity of carcinoids, named the “supra-carcinoids," was unveiled. These samples were characterized by a morphology similar to that of pulmonary carcinoids but with the molecular features of LCNEC samples. In the pan-LNEN TumorMap, the supra-carcinoids also clustered with the LCNEC samples and were molecularly closer to LCNECs than to SCLCs (Wilcoxon *P* = 5 × 10^−2^). We also note that 1 sample from Laddha et al. [8] LC2 cluster (SRR7646258) clusters with LCNEC.

### Quality control of the UMAP projection

In any dimensional reduction technique, there is a trade-off between preserving the global structure of the data and the fine-scale details, and UMAP has been designed to reach a better balance compared with previous methods.

On the basis of the previously published analyses of LNEN data [2, 4–8], we expect the global structure of the data to be composed of 6 molecular groups (SCLCs, Type I and Type II LCNECs, Carcinoid A1, A2, and B). For this reason, an ideal projection able to capture this large-scale variation should contain 5 dimensions. To assess the quality of the 2D representation generated by UMAP, we propose a comparative analysis between UMAP and the traditional PCA based on the 5 first principal components of PCA (PCA-5D) as implemented in the dudi.pca function from the ade4 R package (v1.7-15) [46]. Because UMAP is aiming at preserving the global structure in only 2 dimensions, we also compared it to the traditional PCA based only on the 2 first principal components (PCA-2D). We evaluated the performance of the methods on the basis of the preservation of (i) the samples’ neighborhood and (ii) the spatial auto-correlations.

#### Preservation of the samples’ neighborhood

We used the sequence difference view (SD) metric (eq. 3 from [47]) to evaluate the preservation of the samples’ neighborhood. This dissimilarity metric compares, for a given sample, its neighborhood in the low-dimensional space with that in the original space, taking into account that preserving the rank of a close neighbor is more important than for a distant neighbor (see [47] for details). SD values are positive ($\mathrm{SD}\in \mathopen {[}0\, ;+\infty \mathclose {)}$), with small values indicating a good preservation of the sample neighborhood. We denote by $\overline{SD}_{k}$ the value of SD averaged across samples for a fixed number of neighbors *k*; $\overline{\mathrm{SD}}_{k}$ gives a sense of the overall preservation of the neighborhood at different scales: local for low *k* values and global for large *k* values. We calculated $\overline{\mathrm{SD}}_{k}$ for PCA-5D, PCA-2D, UMAP with n_neighbors = 238, and UMAP with the default value n_neighbors = 15. Because we are interested in the relative values of $\overline{\mathrm{SD}}_{k}$ for the different dimensionality reduction methods, and because we use PCA as a reference, for each dimensionality reduction method *X* we scaled the values of $\overline{\mathrm{SD}}_{k}$ using that of PCA-5D and PCA-2D:

(1) \begin{eqnarray*}
\overline{\mathrm{SD}}_{k,X}^{\prime } = \frac{ \overline{\mathrm{SD}}_{k,X} - \overline{\mathrm{SD}}{_{k,\mathrm{PCA-5D}}}}{\overline{\mathrm{SD}}{_{k,\mathrm{PCA-2D}}} - \overline{\mathrm{SD}}{_{k,\mathrm{PCA-5D}}}}. \end{eqnarray*}

By definition, $\overline{\mathrm{SD}}_{k,\mathrm{PCA-5D}}^{\prime }=0$ and $\overline{\mathrm{SD}}_{k,\mathrm{PCA-2D}}^{\prime }=1$. Thus values of $\overline{\mathrm{SD}}_{k,X}^{\prime }$ close to 0 indicate that *X* preserves *k* neighborhoods as well as PCA-5D, whereas values close to 1 indicate that *X* preserves *k* neighborhoods worse than PCA-5D but as well as PCA-2D, and values >1 indicate that *X* preserves *k* neighborhoods worse than PCA-2D and PCA-5D. Note that $\overline{\mathrm{SD}}_{k,X}^{\prime }$ can be negative if *X* preserves *k* neighborhoods better than $\overline{\mathrm{SD}}{_{k,\mathrm{PCA-5D}}}$. For the UMAP projection, we iterated the computation of $\overline{\mathrm{SD}}_{k}^{\prime }$ 1,000 times because the algorithm uses a stochastic optimization step to define the projection.

**Figure 4:**
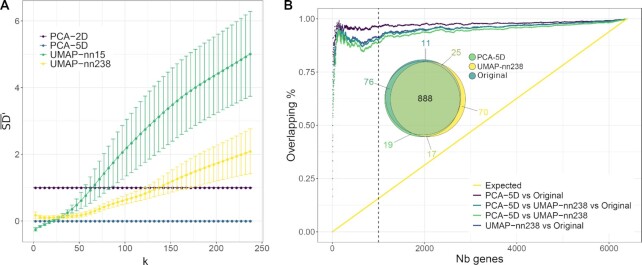
Quality controls performed on the UMAP projection. (**A)** Comparison of the samples’ neighborhood preservation for UMAP, PCA-2D, and PCA-5D dimensionality reductions. $\overline{\mathrm{SD}}_{k}^{\prime }$ values are represented as a function of the number *k* of nearest neighbors considered, for different dimensionality reduction methods: PCA-2D in purple, PCA-5D in blue, UMAP with n_neighbors = 238 (UMAP-nn-238) in yellow, and UMAP with the default value n_neighbors = 15 (UMAP-nn-15) in green. Error bars correspond to the means ± standard deviations computed across 1,000 replicate simulations. (**B)** Concordance between gene expressions’ spatial auto-correlations in the original space, UMAP-nn-238, and PCA-5D dimensionality reductions. For each space, the genes were ranked on the basis of the spatial auto-correlations of their expression (mean MI values). The concordance is measured as the proportion of overlap between the top *N* genes in the different spaces (colored lines). The yellow line corresponds to the proportion of overlap expected under the null hypothesis (based on the expected mean of the hypergeometric law). The Euler diagram represents the overlaps between the top 1,000 features (*N* = 1,000, dashed line) resulting from the 3 spaces.

As expected, increasing the n_neighbors UMAP parameter from 15 to 238 leads to a better preservation of the global structure, clearly visible for *k* > 30 (Fig. 4A; mean $\overline{\mathrm{SD}}_{k> 30}^{\prime }$ = 2.855 and 1.029, respectively), while only marginally reducing the preservation of the local structure for *k* < 30 (mean $\overline{\mathrm{SD}}_{k< 30}^{\prime }$ = −0.076 and 0.124, respectively), which is approximately the size of the smallest cluster. Globally, the $\overline{\mathrm{SD}}_{k}^{\prime }$ values over all *k* levels are lower for an n_neighbors value of 238 than 15 (paired *t*-test *P* = 6.09 × 10^−8^). With n_neighbors = 238, the UMAP projection provides a clear improvement over PCA-2D for *k* ∼ 135 (mean $\overline{\mathrm{SD}}_{k}^{\prime }< 1$), offering a good trade-off for visualization in only 2 dimensions while being able to maintain the global structure of the data, in particular the 6 molecular groups previously identified. This observation highlights the importance of varying the n_neighbors parameter according to the purpose of the projection. Some analyses would require the local structure of the sample neighborhood to be maintained, while others, the global structure.

#### Preservation of spatial auto-correlations

Under the hypothesis that close points on projections share a similar molecular profile, spatial auto-correlations were measured according to the Moran index (MI) metric [48]. MI values range from −1 to 1, the extreme values indicating negative (nearby locations have dissimilar gene expression) or positive (nearby locations have similar gene expression) spatial auto-correlation, respectively. The spatial auto-correlation of the expression of each gene helps to identify the genes contributing to the structure of the molecular map (MI ≃ 1), and conversely, the genes that are randomly distributed spatially (MI ≃ 0). The computation of MI requires a weight matrix that determines the spatial scale at which auto-correlation is assessed; we gave a weight of 1 to the *k* nearest neighbors based on Euclidean distance, and 0 otherwise, so that we can control the scale at which MI is computed with parameter *k*. The mean MI across *k* values was computed for all gene expression features for: (i) the original space, (ii) the PCA-5D projection, and (iii) the UMAP projection (with n_neighbors = 238). We used the implementation of MI from the Moran.I function of R package ape (v. 5.3) [49].

To evaluate the preservation of the spatial auto-correlations, we ranked the top *N* genes based on the mean MI values for these 3 cases and calculated the overlap between the lists (Fig. 4B). We found that the PCA-5D is only slightly more conservative of the spatial auto-correlations found in the original space than UMAP (unilateral paired *t*-test *P* = 2.2 × 10^−16^). For example, for *N* = 1,000 (see Euler diagram inserted in Fig. 4B), 88.8% of the genes with the highest MI overlap between the PCA-5D, UMAP, and the original space.

## Reuse potential

### An interactive TumorMap

Newton and colleagues have recently developed a portal called TumorMap [13,50], an online tool dedicated to omics data visualization. This new type of integrated genomics portal uses the Google Maps technology designed to facilitate visualization, exploration, and basic statistical interrogation of high dimensional and complex datasets. The pan-LNEN molecular map that we generated in this work (Fig. 3) has been shared on the TumorMap platform. Along with the molecular map, the main clinical, histopathological and molecular features highlighted in the previous studies were uploaded as attributes. The interface enables users to explore and navigate through the map: zooming in and out, coloring and filtering samples based on attributes. The users can also create their own attributes based on pre-existing ones by using operators such as union or intersection. In addition, multiple statistical tests are pre-implemented and available, for example: comparison of attributes without considering the samples positions on the map, comparison of attributes considering samples' positions on the map, and ordering attributes on the basis of their potential to differentiate 2 groups of samples. The interactive nature of the map and the fact that its manipulation does not require computational expertise, could enable the generation of new hypotheses and expand the reuse potential of the dataset.

### An interactive computational notebook

In the first part of the article, we described the pre-processing and QC steps applied on the recently published LNEN multi-omics dataset [7] in order to facilitate its reuse. To generate the pan-LNEN molecular map, the same pre-processing steps were followed to homogenize independently published transcriptomic data [2,4–8]. For that purpose, reproducible pipelines, developed in house, were used and are available for reuse to the scientific community on GitHub [51] (see the “data description” section). In addition, the code used to generate the molecular map and to evaluate the quality of the dimensionality reduction is provided as a notebook published on Nextjournal [52]. Along with the code, the notebook provides the data and the dependencies required to run the analyses performed in this paper. Interested researchers can thus make a copy of this publicly available notebook (called “Remix”) to reproduce our results but also interactively modify the code and explore the influence of different parameters.

### Integration of new samples

The homogenized read counts of the pan-LNEN data are available on GitHub [14]. Along with the available code, these data could be used to integrate new samples for which RNA-Seq data are available. The raw read counts of the new samples should firstly be generated following the same processing steps described in the section “Data quality controls” (Fig. 1, middle panel) and integrated to the pan-LNEN read counts. We also provide in the Nextjournal notebook, the Nextflow command lines allowing to obtain the read counts. The vst (DESeq2 [39]) should then be applied on the combined dataset and UMAP should finally be rerun to project all samples together in a 2D space. All together, we provide the resources to integrate additional samples into our molecular map, starting from raw sequencing read counts.

## Discussion

Genomic projects focused on rare cancers encounter the limitation of availability of high-quality biological material suitable for such studies. This translates in small series of samples usually underpowered to draw meaningful conclusions. Thus, tools facilitating the integration of independent datasets into larger sample series will lead to more informative studies. Recently, the first multi-omic dataset for the understudied atypical pulmonary carcinoids and the first methylation dataset for LCNECs was published [7]. Here we provide a parallel description of the pre-processing of these molecular data and provide evidence of the good quality of the different ’omics data generated. This data collection associated with previous datasets [2,4–6, 8] completes the LNEN molecular landscape and thus provides a valuable resource to improve the molecular characterization of LNEN tumors. Notably, we show here the perfect concordance of the 3 molecular clusters of pulmonary carcinoids independently identified in [7] and [8], validating the discoveries made by these 2 studies and proving the usefulness of this integrative approach.

However, even when primary genomic data are available, barriers to accessing the data still exist, often limiting reuse by the community [53]. In particular, downloading and re-reprocessing large raw sequencing datasets requires dedicated infrastructure and bioinformatics skills. Indeed, to minimize batch effects when integrating data from different studies, one needs to process it in exactly the same way (e.g., with the same versions of the same software, the same reference genome, the same annotation databases). As more and more data are generated, the previously mentioned reprocessing will become untenable and replicating these efforts for each new study in each research group represents a waste of resources. Standardization of laboratory and computational protocols might become a reality when large national medical genomics initiatives become fully operational [54]. In the meantime there is a need for better data sharing strategies than the traditional “supplementary spreadsheet/raw data” combination that can accelerate the translational impact of molecular findings.

One step in this direction is the generation of so-called “tumor maps,” which provide an interactive way to explore the molecular data and allow easy statistical interrogation, including generating new hypotheses, but also projecting data from future studies including fewer samples [13]. This integration method has some limitations though. A fixed reference map could be of interest for easier biological interpretations, but the overall sample size of the datasets used to build the pan-LNEN map remains relatively small. Thus, the map probably does not capture the complete molecular diversity of the LNENs, and integrating new samples will influence the map and potentially change the clusters obtained after dimensionality reduction. Also, if the harmonization of the new dataset to integrate is not enough to correct for strong batch effects, the interpretation of the projections would be erroneous. Another approach would be to project the new samples into a fixed reference map. However, the stochastic nature of UMAP embedding and its sensitivity to parameter tuning can lead to unstable projection results; thus this task is for now not straightforward and requires further development [55]. In the meantime, favoring the integration of datasets will, over the years, yield the constitution of molecular maps that will probably be more and more accurate and more adapted to the projection of new samples.

## Conclusion

Here we provide a molecular map based on homogenized transcriptomic data available for the 4 types of LNENs from 6 different studies. We show that this map represents well both the local and global structure of the data and captures the main biological features previously reported. We provide a full spectrum of data and tools to maximize reuse potential for a wide range of users: raw sequencing reads, gene expression matrix, bioinformatics pipelines, interactive computational notebooks, and an interactive TumorMap. In particular, we indicate how one can update the molecular map by integrating new samples starting from raw sequencing reads. Considering the small sample sizes of molecular studies on rare LNENs, promoting data integration will empower more reliable statistical testing, and this map will therefore serve as a reference in future studies.

## Availability of Supporting Data and Materials

R codes used for this article are available in the GigaDB data repository [56]. The data used in this manuscript are available in the European Genome-phenome Archive (EGA), which is hosted at the EBI and the Centre for Genomic Regulation (CRG), under the accession numbers EGAS00001003699, EGAS00001000650, EGAS00001000925, EGAS00001000708, as well as on Gene expression Omnibus (GEO) under GEO SuperSeries GSE118131.

## Ethical Approval

These data belong to the lungNENomics project, which has been approved by the IARC Ethical Committee.

## Additional Files

Supplementary Table 1: Sample overview

Supplementary Table 2: Summary table of STAR metrics

Supplementary Table 3: Summary table of RSeQC metrics

Supplementary Table 4: Summary table of HTSeq metrics

Supplementary Table 5: List of filtered probes

Supplementary Table 6: Sample methylation metadata

## Abbreviations

AC: atypical carcinoids; ABRA: Assembly-Based Realigner; BAM: Binary Alignment Map; CDS: coding sequence; CGR: Center for Genomic Regulation; CpG: cytosine–phosphate–guanine; CTAT: Trinity Cancer Transcriptome Analysis Toolkit; dbSNP: Single Nucleotide Polymorphism Database; EGA: European Genome-phenome Archive; EMBL-EBI: European Bioinformatics Institute; GATK: Genome Analysis Toolkit; IARC: International Agency for Research on Cancer; LCNEC: large-cell neuroendocrine carcinoma; LCNEC/SCLC-like: large-cell neuroendocrine carcinomas with the molecular features of small cell lung cancers; LNEN: lung neuroendocrine neoplasm; Mb: megabase pairs; MI: Moran index; NEC: neuroendocrine carcinoma; NEN: neuroendocrine neoplasm; NET: neuroendocrine tumor; PCA: principal component analysis; QC: quality control; RNA-Seq: RNA sequencing; SCLC: small-cell lung cancer; SCLC/LCNEC-like: small-cell lung cancers with the molecular features of large-cell neuroendocrine carcinomas; SCLC/SCLC-like: small-cell lung cancers with the molecular features of small-cell lung cancers; SD: Sequence Difference view metric; SNP: single-nucleotide polymorphism; STAR: Spliced Transcripts Alignment to a Reference; TC: typical carcinoids; TES: transcription end site; TSS: transcription start site; UCSC: University of California Santa Cruz; UMAP: Uniform Manifold Approximation and Projection; vst: variance-stabilized transformation; WES: whole-exome sequencing; WGS: whole-genome sequencing.

## Competing Interests

The authors declare no conflict of interest. Where authors are identified as personnel of the International Agency for Research on Cancer/World Health Organization, the authors alone are responsible for the views expressed in this article and they do not necessarily represent the decisions, policy or views of the International Agency for Research on Cancer/World Health Organization.

## Funding

This work has been funded by the US National Institutes of Health (NIH R03CA195253 to L.F.C. and J.D.M.), the French National Cancer Institute (INCa, PRT-K-17-047 to L.F.C. and M.F.), the Ligue Nationale contre le Cancer (LNCC 2016 to L.F.C.), France Genomique (to J.D.M.), and the Neuroendocrine Tumor Research Foundation (NETRF, Investigator Award 2019 to L.F.C.). L.M. has a fellowship from the LNCC.

## Authors' Contributions

M.F. and L.F.C. conceived and designed the study. A.A.G.G., E.M., N.A., L.M., and C.V. performed the analyses. V.C. and A.G. gave scientific input for the methylation part. J.D.M. helped with logistics and gave scientific input. A.A.G.G., E.M., N.A., M.F., and L.F.C. wrote the manuscript. All the authors read and commented the manuscript.

## Supplementary Material

giaa112_GIGA-D-20-00021_Original_Submission

giaa112_GIGA-D-20-00021_Revision_1

giaa112_GIGA-D-20-00021_Revision_2

giaa112_Response_to_Reviewer_Comments_Original_Submission

giaa112_Response_to_Reviewer_Comments_Revision_1

giaa112_Reviewer_1_Report_Original_Submission Fuqiang Li -- 2/25/2020 Reviewed

giaa112_Reviewer_2_Report_Original_Submission Saurabh V Laddha -- 3/11/2020 Reviewed

giaa112_Supplemental_Tables
