# Supplementary material for: A molecular map of lung neuroendocrine neoplasms
Source: Gigascience. 2020 Oct 30;9(11):giaa112. doi: 10.1093/gigascience/giaa112 (PMC7596803; doi:10.1093/gigascience/giaa112)
Supplement: giaa112_GIGA-D-20-00021_Revision_1 [file giaa112_giga-d-20-00021_revision_1.pdf]

GigaScience

A molecular map of lung neuroendocrine neoplasms

--Manuscript Draft--

**Manuscript Number:**	GIGA-D-20-00021R1
**Full Title:**	A molecular map of lung neuroendocrine neoplasms
**Article Type:**	Data Note
**Funding Information:**	National Institutes of Health (R03CA195253) — Dr. Lynnette Fernandez-Cuesta<br>Institut National Du Cancer (PRT-K-17-047) — Dr. Lynnette Fernandez-Cuesta<br>Ligue Contre le Cancer (LNCC-2016) — Dr. Lynnette Fernandez-Cuesta<br>France Genomique (FR) — Dr. James D McKay<br>Neuroendocrine Tumor Research Foundation (Investigator Award 2019) — Dr. Lynnette Fernandez-Cuesta<br>Ligue Contre le Cancer — Ms. Lise Mangiante
**Abstract:**	Background Lung neuroendocrine neoplasms (NENs) are rare solid cancers, with most genomic studies including a limited number of samples. Recently, generating the first multi-omic dataset for atypical pulmonary carcinoids and the first methylation dataset for large-cell neuroendocrine carcinomas (LCNEC) led us to the discovery of clinically relevant molecular groups as well as a new entity of pulmonary carcinoids (supra-carcinoids). Results In order to promote the integration of lung NENs molecular data, we provide here detailed information on data generation and quality control for whole genome/exome sequencing, RNA sequencing, and EPIC 850k methylation arrays for a total of 84 lung NENs patients. We integrate the transcriptomic data with other previously published data and generate the first comprehensive molecular map of lung NENs using the Uniform Manifold Approximation and Projection (UMAP) dimension reduction technique. We show that this map captures the main biological findings of previous studies and can be used as reference to integrate datasets for which RNA sequencing is available. The generated map can be interactively explored and interrogated on the UCSC TumorMap portal (https://tumormap.ucsc.edu/?p=RCG_lungNENomics/LNEN). The data, source code, and compute environments used to generate and evaluate the map as well as the raw data are available respectively in a Nextjournal interactive notebook (https://nextjournal.com/rarecancersgenomics/a-molecular-map-of-lung-neuroendocrine-neoplasms/), and at the EMBL-EBI European Genome-phenome Archive and Gene Expression Omnibus data repositories. Conclusions We provide data and all resources needed to integrate it with future lung NENs transcriptomic studies, allowing to draw meaningful conclusions that will eventually lead to a better understanding of this rare understudied disease.
**Corresponding Author:**	Matthieu Foll<br><br>FRANCE
**Corresponding Author Secondary Information:**	
**Corresponding Author's Institution:**	
**Corresponding Author's Secondary Institution:**	
**First Author:**	Aurélie AG Gabriel
**First Author Secondary Information:**	
**Order of Authors:**	Aurélie AG Gabriel<br>Emilie Mathian

Powered by Editorial Manager® and ProduXion Manager® from Aries Systems Corporation

	Lise Mangiante
	Catherine Voegele
	Vincent Cahais
	Akram Ghantous
	James D McKay
	Nicolas Alcala
	Lynnette Fernandez-Cuesta
	Matthieu Foll
**Order of Authors Secondary Information:**	
**Response to Reviewers:**	Dear Editor,<br><br>Pleased find enclosed the revised version of our manuscript as well as a a point-by-point response to the reviewers' comments.<br><br>We have addressed all the points raised by the reviewers, and importantly, we have integrated the additional dataset proposed by reviewer 2. We are very pleased to see that this new data reinforces our conclusions and thank the reviewer for the suggestion.<br><br>Note that we have generated an annotated pdf file to detail and highlight the changes made for this review that can be found as a supplementary file.<br><br>Yours sincerely,<br><br>Matthieu Foll<br><br><br>Reviewer #1: The authors provide detailed information of multi-omic dataset for a total of 84 lung NENs patients and molecular map integrated with other studies. The high quality multi-omic dataset provided here could help break the study of lung NENs with limited number samples. The detailed process and quality control of generation of an integrative molecular map could help other users to re-use the dataset.<br>Carrying out open, named peer review I was able to get access to and inspect the data via the IARC Data Access Committee (DAC). Upon applying I got a response from the IARC DAC within a few hours. I was asked to sign a Data Access Agreement before they released the raw data via the EBI EGA. The EGA helpdesk then released the data to my account within 3 days. The data was organized in three datasets according to data type, and users could easily understand what was there. Upon inspection the data stored in the EGA was in accordance with what the authors describe in their accompanying Data Note manuscript.<br><br>-> We thank the reviewer for the smooth process during the data access request and signing the required data access agreement.<br><br>Minor Revisions:<br>1. The software used for indel local realignment in manuscript is 'ABRA', the same as the published paper 'Integrative and comparative genomic analyses identify clinically relevant pulmonary carcinoid groups and unveil the supra-carcinoids' , while in pipeline (https://github.com/IARCbioinfo/alignment-nf) is GATK.<br><br>-> The option --indel_realignment was not used when running the pipeline alignment-nf on the WES data. Instead, ABRA was run independently afterwards. To clarify this step, we added a sentence in the manuscript page 2 in the paragraph “WES and WGS data“.<br><br>2. A brief introduction of some basic information (e.g. sequencing platform, exome kits) of sequencing data would be helpful.<br><br>-> Sequencing information for each omic dataset has been added in the manuscript,

Powered by Editorial Manager® and ProduXion Manager® from Aries Systems Corporation

	page 2 and page 3 in the paragraphs “WES and WGS data” and “RNA-Seq data” respectively.<br><br>3. A table with samples (row) and omic data (column) whether present (Yes or No) would be helpful.<br><br>-> A supplementary table (Supplementary table 1) has been added, this table contains for each omic a column specifying if the omic data is available or not for each sample. In the text, the table is referenced page 2 in the section “Data quality controls”.<br><br>4. A table with clinical information would be helpful too.<br>Clinical data such as age, sex, survival and smoking status have been added to the Supplementary Table 1 previously mentioned.<br><br>Reviewer #2: The study is well written and present interesting Omics dataset for lung neuroendocrine tumors(LNETs). As these tumors are rare in nature, it is important to create such resources for researcher.<br>Minor Revisions:<br>- Using genomics datasets, the authors should able to comment on the tumor purity and provide a table. This is also one important data note for this cancer type.<br><br>-> When available, tumor content information has been added to the Supplementary Table 1 previously mentioned.<br><br>- Chan CS et al., (PMID:31300474) recently found three novel molecular subtypes of Lung Carcinoids. Authors should consider this dataset and integrate with all lung dataset to enrich for sample size and also one shop center for this cancer type for researcher.<br><br>-> We thank the reviewer for pointing us to this interesting additional dataset that we missed. We have now integrated it to our pan-LNEN map and the number of carcinoids considered in the molecular map increased from 88 to 118. Very interestingly this additional dataset allowed us to confirm the existence of three molecular clusters previously identified independently by Alcala et al. and Laddha et al. in 2019. Indeed the clusters LC1, LC2 and LC3 identified by Laddha et al. perfectly matched respectively the cluster A1, B and A2 from Alcala et al. As we had additional data to reprocess, we also took the opportunity to update some software/reference versions at the same time and reprocessed all transcriptomes. Figure 1 middle panel, paragraph “RNA-Seq data” page 3 and 4, Figure 2D and the section “Availability of source code” page 8 have been modified accordingly. Also, the results associated with the molecular map, presented in the section “Generation of an integrative molecular map” have been updated. Finally, the resources complementing this paper, the nextjournal notebook and the DRMetrics GitHub repository have been updated.
**Additional Information:**	
**Question**	**Response**
Are you submitting this manuscript to a special series or article collection?	No
**Experimental design and statistics**<br><br>Full details of the experimental design and statistical methods used should be given in the Methods section, as detailed in our Minimum Standards Reporting Checklist. Information essential to interpreting the data presented should be made available in the figure legends.	Yes

Powered by Editorial Manager® and ProduXion Manager® from Aries Systems Corporation

Have you included all the information requested in your manuscript?	
**Resources**<br><br>A description of all resources used, including antibodies, cell lines, animals and software tools, with enough information to allow them to be uniquely identified, should be included in the Methods section. Authors are strongly encouraged to cite Research Resource Identifiers (RRIDs) for antibodies, model organisms and tools, where possible.<br><br>Have you included the information requested as detailed in our Minimum Standards Reporting Checklist?	Yes
**Availability of data and materials**<br><br>All datasets and code on which the conclusions of the paper rely must be either included in your submission or deposited in publicly available repositories (where available and ethically appropriate), referencing such data using a unique identifier in the references and in the "Availability of Data and Materials" section of your manuscript.<br><br>Have you have met the above requirement as detailed in our Minimum Standards Reporting Checklist?	Yes

Powered by Editorial Manager® and ProduXion Manager® from Aries Systems Corporation

Manuscript

Click here to access/download;Manuscript;Gigascience_paper.pdf

OXFORD (GIGA)^n SCIENCE

GigaScience, 2020, 1–10

doi: xx.xxxx/xxxx
Manuscript in Preparation
Paper

PAPER

A molecular map of lung neuroendocrine neoplasms.

Aurélie AG Gabriel[1,†], Emilie Mathian[1,†], Lise Mangiante[1], Catherine Voegele[1], Vincent Cahais[2], Akram Ghantous[2], James D McKay[1], Nicolas Alcala[1], Lynnette Fernandez-Cuesta[1,‡] and Matthieu Foll[1,*,‡]

[1]Section of Genetics, International Agency for Research on Cancer (IARC-WHO), Lyon, France and [2]Section of Mechanisms of Carcinogenesis, International Agency for Research on Cancer (IARC-WHO), Lyon, France

[*]**Correspondence address.** Matthieu Foll, International Agency for Research on Cancer, 150 cours Albert Thomas, 69372 Lyon CEDEX 08, France. E-mail: follm@iarc.fr
[†]Contributed equally.
[‡]Jointly supervised.

Abstract

Background Lung neuroendocrine neoplasms (NENs) are rare solid cancers, with most genomic studies including a limited number of samples. Recently, generating the first multi-omic dataset for atypical pulmonary carcinoids and the first methylation dataset for large-cell neuroendocrine carcinomas (LCNEC) led us to the discovery of clinically relevant molecular groups as well as a new entity of pulmonary carcinoids (supra-carcinoids). **Results** In order to promote the integration of lung NENs molecular data, we provide here detailed information on data generation and quality control for whole genome/exome sequencing, RNA sequencing, and EPIC 850k methylation arrays for a total of 84 lung NENs patients. We integrate the transcriptomic data with other previously published data and generate the first comprehensive molecular map of lung NENs using the Uniform Manifold Approximation and Projection (UMAP) dimension reduction technique. We show that this map captures the main biological findings of previous studies and can be used as reference to integrate datasets for which RNA sequencing is available. The generated map can be interactively explored and interrogated on the UCSC TumorMap portal (https://tumormap.ucsc.edu/?p=RCG_lungNENomics/LNEN). The data, source code, and compute environments used to generate and evaluate the map as well as the raw data are available respectively in a Nextjournal interactive notebook (https://nextjournal.com/rarecancersgenomics/a-molecular-map-of-lung-neuroendocrine-neoplasms/), and at the EMBL-EBI European Genome-phenome Archive and Gene Expression Omnibus data repositories. **Conclusions** We provide data and all resources needed to integrate it with future lung NENs transcriptomic studies, allowing to draw meaningful conclusions that will eventually lead to a better understanding of this rare understudied disease.

Key words: Carcinoids, lung cancer, neuroendocrine neoplasms, rare cancers, genomics, Tumormap, lungNENomics project

Background

Lung neuroendocrine neoplasms (lung NENs or LNENs) are rare understudied diseases with limited therapeutic opportunities. Lung NENs include poorly differentiated and highly aggressive lung neuroendocrine carcinomas (NECs)–i.e., small-cell lung cancer (SCLC) and large-cell neuroendocrine carcinoma (LCNEC)–as well as well-differentiated and less aggressive lung neuroendocrine tumors (NETs)–i.e., typical and atypical carcinoids (WHO classification 2015 [1]). Over the past years several genomic studies have investigated the molecular characteristics of these diseases in order to provide some evidence for a more personalized clinical management [2, 3, 4, 5, 6, 7, 8]. Although lung NECs and NETs are broadly considered

Compiled on: June 4, 2020.
Draft manuscript prepared by the author.

as different diseases, several recent studies have suggested that they may share some molecular characteristics [9, 10, 7, 11, 12]. However, due to the rarity of these diseases, the sample sizes of these studies individually are limited, and the integration of independent datasets is not an easy task.

Providing a way to interactively visualize and analyze these pan-LNEN data would be of great interest for the scientific community, not only to further explore the proposed molecular link between lung NECs and NETs, but also to integrate data from studies including fewer samples to reach the statistical power needed to draw meaningful conclusions.

Data Description

Recently [7], we performed the first integrative and comparative genomic analysis of lung NEN samples from all histological types, based on newly sequenced data: whole-exome data (WES, 16 samples), whole-genome data (WGS, 3 samples), RNA-Seq data (20 samples), and EPIC 850K methylation data (76 samples), as well as publicly available data. These data correspond to the most extensive multi-omic dataset of lung NENs, including the first methylation data for LCNEC and the first molecular characterization of the rarest lung NEN subtype (atypical carcinoids) [7]. This dataset, which provides the missing pieces for a complete molecular characterization of lung NENs, have been deposited at the EMBL-EBI European Genome-phenome Archive (EGA accession number EGAS00001003699). In order to facilitate the reuse of the data generated in the previous manuscript [7], we provide here a complementary data descriptor by outlining the preprocessing and the quality control (QC) steps performed on each omic dataset available on EGA.

Also, other studies have generated sequencing data and performed a molecular characterization of lung NEN samples: pulmonary carcinoids (mostly typical carinoids) have been characterized by Fernandez-Cuesta *et al.* and Laddha *et al.* [4, 8], LCNEC by George *et al.* [6] and SCLC by George *et al.* [5] and Peifer *et al.* [2]. We therefore generate the first pan-LNEN molecular tumor map by integrating the transcriptomic data from Alcala *et al.* [7] and the other published lung NEN transcriptomic data [2, 4, 5, 6, 8]. This map provides an interactive way to explore the molecular data and allows statistical interrogation, based on the UCSC TumorMap portal [13]. The integrated transcriptomic dataset resulting from these studies is available on GitHub [14].

Data quality controls

Figure 1 provides a schematic view of the preprocessing steps and the associated quality controls performed for each omic dataset generated by Alcala and colleagues [7]. An overview of the available omics and clinical data for each sample is provided in Supplementary Table 1.

WES and WGS data

WES and WGS were performed respectively on 16 and 3 fresh frozen atypical carcinoids in the Cologne Centre for Genomics and the Centre National de Recherche en Génomique Humaine (CNRGH). For WES, the SeqCap EZ v2 Library capture kit from NimbleGen (44Mb) and the Illumina HiSeq 2000 machine (Illumina Inc., CA, USA) were used for the sequencing. For WGS, the Illumina TruSeq DNA PCR-Free Library Preparation Kit was used for library preparation and the HiSeqX5 platform from Illumina for the sequencing as described in [7]. The sequencing reads from the 16 atypical carcinoids whole-exomes and the 3 carcinoids whole-genomes were processed using the in-house Nextflow [15] workflow in the IARCbioinfo/alignment-nf GitHub repository (see Code Availability section) consisting in three steps: mapping reads to the reference genome (GRCh37), marking duplicates and sorting reads, using bwa (v0.7.12-r1044) [16], samblaster (v0.1.22) [17], and sambamba (v0.5.9) [18] respectively. For WES samples, local realignment using ABRA (v0.97b [19]) was then run. The quality controls of the WES and WGS data were performed using FastQC v0.11.8 [20] and QualiMap v2.2.1 [21] and the results aggregated using MultiQC v1.7 [22] (Figure 1, left panel).

Figure 2A-B, show the per base sequence quality scores (left panels) and the per sequence mean quality scores (right panels). Regarding the per base sequence quality scores, the majority of the base calls were of very good quality (>28, green area, Figure 2A left panel) and of reasonable quality (>20, orange area, Figure 2B left panel) for WES and WGS data respectively. The most frequently observed sequence mean quality score was around 30 for both techniques, which is equivalent to an error probability of 0.1%. Table 1 provides the general statistics associated to the WES and WGS quality controls. The observed median coverage for each sample was above the expected coverage (30X for the WGS samples and 120X for the WES samples). Concerning the alignment quality, all WES samples had more than 99% of the reads aligned and all WGS samples had more than 98% of the reads aligned.

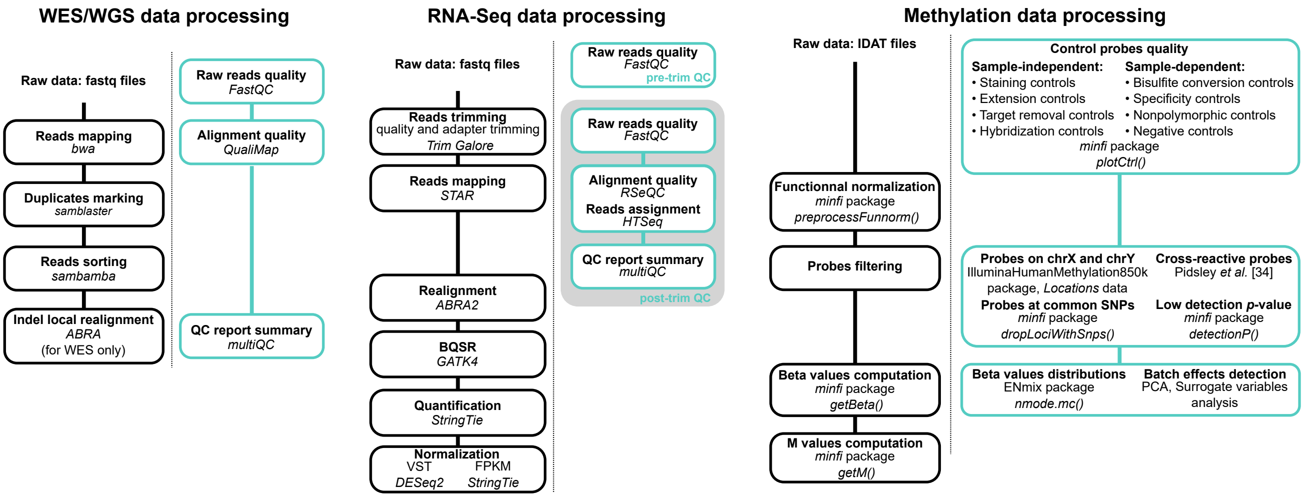

Figure 1. Bioinformatics workflows for data processing and associated quality controls. Bioinformatics tools used for the processing of the WES/WGS data, RNA-Seq and methylation data are represented in the left, middle and right panels respectively. Green boxes correspond to quality controls (QC) steps.

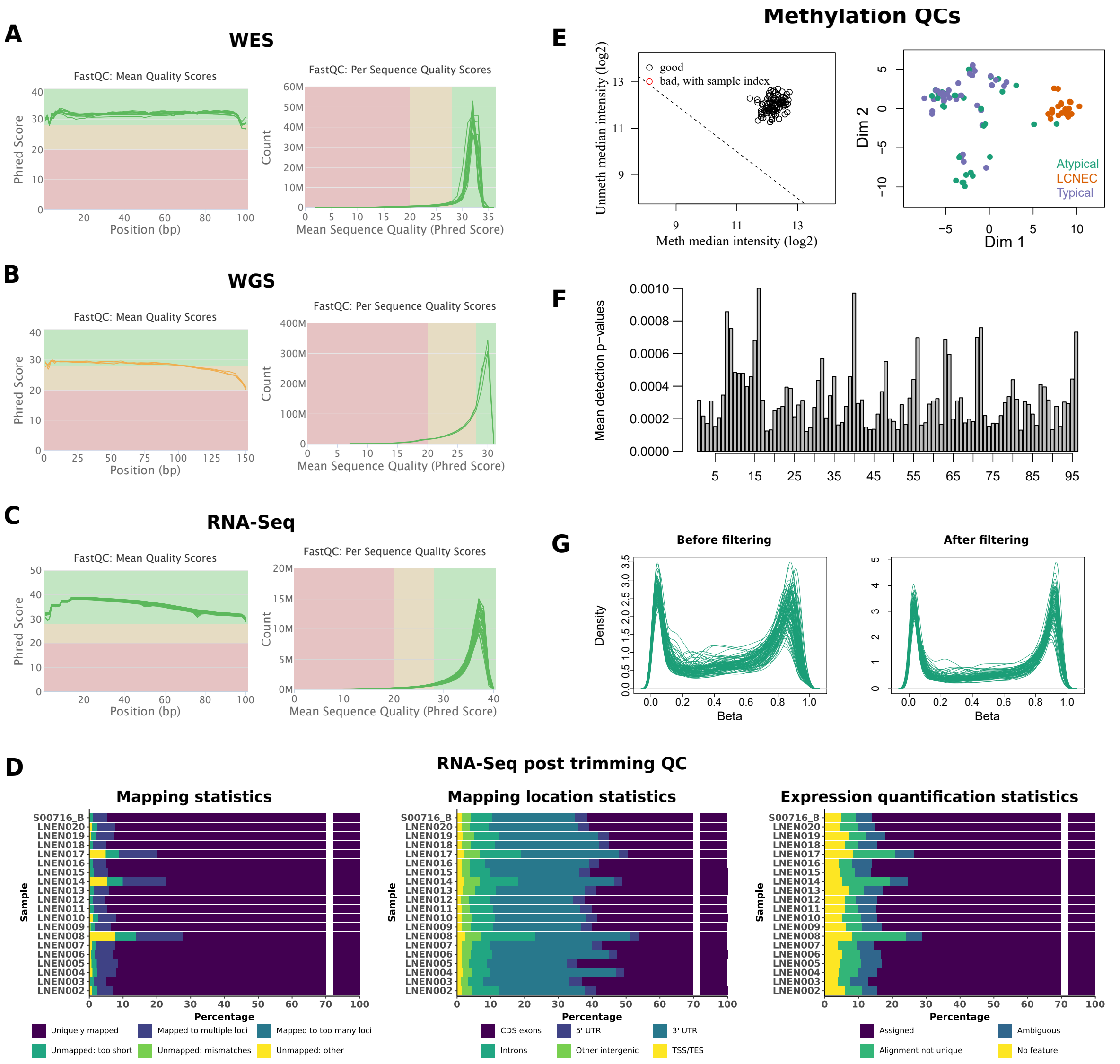

Figure 2. Quality controls performed on each omic dataset. A) Reads quality control using FastQC for WES data. **B)** Reads quality control using FastQC for WGS data. **C)** Reads quality control using FastQC for RNA-Seq data. For A, B, and C, the left panels correspond to the sequence quality plots, the x-axis representing the base position in the read and the y-axis the mean quality value; the right panels correspond to the per sequence quality scores plots, the x-axis representing the mean quality score and the y-axis the number of reads. **D)** Quality control of the RNA-Seq data after trimming. Left panel: barplot representing the percentages of reads uniquely mapped ("Uniquely mapped"), mapped to multiple loci ("Mapped to multiple loci" or "Mapped to too many loci" if the number of loci is higher than 10), unmapped because the mapped reads' proportion was too small ("Unmapped: too short"), unmapped because of too many mismatches ("Unmapped: mismatches"), or unmapped for other reasons ("Unmapped: other"). Middle panel: cumulative barplot representing the percentages of reads mapped, using RSeQC, at different locations in the genome (exons, introns, 5' and 3' UTR, intergenic regions, TSS, and TES). Right panel: cumulative barplot representing the cumulative percentages associated to the different reads assignments using HTSeq ("Assigned": reads assigned to one gene, "Ambiguous": reads assigned to multiple overlapping genes, "Aligned not unique": reads assigned to multiple non-overlapping genes, "No Feature": reads assigned to none of the features). **E)** Left panel: samples' quality based on log median intensities. The x-axis and y-axis correspond to the median of log2 methylated and unmethylated intensities, respectively. Right panel: representation of the between-sample similarities based on the two first MDS dimensions. **F)** Histogram of the median detection p-value for each sample. **G)** Distribution of the beta values for each sample before and after the filtering step (left and right panel respectively).

RNA-Seq data

RNA-Sequencing was performed on 20 fresh frozen atypical samples. The Illumina TruSeq RNA sample preparation Kit was used for library preparation and the Illumina TruSeq PE Cluster Kit v3 and the Illumina TruSeq SBS Kit v3-HS kits were used on an Illumina HiSeq 2000 sequencer. The data generated were processed using in-house Nextflow [15] workflows consisting in five steps (Figure 1, middle panel): i) reads trimming using Trim Galore v0.6.5 [23], ii) reads mapping to the reference genome (GRCh38, gencode version 33 from bundle CTAT from 6th April 2020 [24]) using STAR v.2.7.3a [25], iii) realignment of the reads using ABRA2 v2.22 [26], iv) base quality score recalibration using GATK4 v4.0.5.1 [27, 28] and v) gene expression quantification using StringTie v2.1.1 [29]. FastQC v.0.11.9 [20], RSeQC v3.0.1 [30] and HTSeq v0.12.4 [31] were used to control the raw reads quality and assignments.

Figure 2C shows that the base calls, before trimming, are of good quality since all samples have a mean per base sequence quality score higher than 28 (left panel) and for all samples the most frequently observed per sequence mean quality is above 35, corresponding to an error probability of 0.03%, (right panel). None of the samples presented more than 1% of over-represented sequences, which assures a proper library diversity. RSeQC was used to control the alignment quality and to assign mapped reads to different genomic features (coding regions, introns, intergenic regions, TSS, TES). Figure 2D (left panel) shows that every sample had more than 70% of reads

Table 1. General statistics associated to the quality controls of the WES and WGS data

Sample	Sequencing	Median coverage	Total nb reads (M)	>30x (%)	Aligned (%)	GC (%)	Median insert size	Duplicates (%)
LNEN002	WES	148	113.3	95.5	99.7	53.7	194	13.9
LNEN003	WES	146	110.3	95.8	99.7	53.7	194	13.4
LNEN004	WES	150	115.3	95.4	99.8	54.3	193	13.1
LNEN005	WES	135	103.4	94.7	99.8	54	195	12.1
LNEN006	WES	126	93.6	94.6	99.8	53.5	197	12.5
LNEN007	WES	145	116.3	94.4	99.8	54.5	195	14.8
LNEN009	WES	123	98.4	92.9	99.7	54.1	195	12.4
LNEN010	WES	138	104.1	95	99.7	53.3	196	13.4
LNEN011	WES	161	125.8	95.8	99.8	54.3	196	14.8
LNEN013	WES	131	99.2	94.3	99.8	53.5	193	13
LNEN014	WES	132	102.6	94	99.8	54.1	195	13.3
LNEN015	WES	148	111.3	95.7	99.6	54.1	197	10.1
LNEN016	WES	133	98	94.3	99.6	54.3	194	9
LNEN017	WES	158	116.4	95.9	99.6	54.1	192	8.9
LNEN020	WES	187	144.7	96.6	99.7	53.6	192	14.5
S00716_B	WES	133	99.8	95.4	99.7	52.8	194	14.3
LNEN041	WGS	36	923.5	77.5	98.9	41	366	13.3
LNEN042	WGS	41	993.7	88.1	98.8	41.5	388	9.4
LNEN043	WGS	43	1033.1	89.7	99.3	41.6	392	8.8

uniquely mapped and the reads distribution for each sample is represented on Figure 2D (middle panel). All samples had more than 75% reads mapped in coding regions (CDS-exons, 5' and 3' UTR exons). The reads counting was performed at the gene level for 59,607 genes (genecode annotation, release 33) using HTSeq [31]. Figure 2D (right panel) shows the reads assignments, the percentage of assigned reads ranges from 71.3 to 87.3%. STAR, RSeQC and HTSeq metrics for each sample are provided in Supplementary Tables 2-4. Note that three samples, LNEN008, LNEN014 and LNEN017, have a higher proportion of reads classified as "Unmapped too short" and "Mapped to multiple loci" (Figure 2D, left panel), reads mapped in intronic regions (Figure 2D, middle panel) and a lower proportion of reads assigned by HTSeq (Figure 2D, right panel) in comparison to the other samples. Unexpected results concerning those samples should be thus considered with caution.

Finally, in order to apply dimensionality reduction methods to the RNA-Seq data (see below), the DESeq2 package v1.26.0 [32] was used to transform the read counts obtained using StringTie to variance stabilized read counts (vst), enabling the comparison of samples with different library sizes. To reduce sex influence on expression profiles, the genes located on sex chromosomes were not considered for subsequent analyses. Genes located on mitochondria chromosomes were as well not considered.

Methylation data

The methylation analyses were performed based on the EPIC 850K methylation arrays and the Infinium EPIC DNA methylation beadchip platform (Illumina) for 33 typical carcinoids, 23 atypical carcinoids, 20 LCNEC and 19 technical replicates in total. These arrays interrogate more than 850,000 CpGs and contain internal control probes that can be used to assess the overall efficiency of the sample preparation steps. The raw intensity data (IDAT files) were processed using the R package *minfi* (v.1.24.0) [33]. Figure 1 (right panel) provides the packages, functions and publication used for the data processing, quality control and filtering steps.

Figure 2E shows that no outliers were detected: i) the left panel, representing the median log2 of the methylated and unmethylated intensities, indicates that all samples cluster together with a log median intensity above 11 for both channels, which supports the absence of failed samples, ii) on the right panel, the multidimensional scaling (MDS) plot shows that the samples cluster together by histological groups. We used the *depectionP* function (*minfi* package), which compares the DNA signal to the background signal based on the negative control probes to provide a detection *p-value* per probe, lower *p-value* indicating reliable CpGs. Figure 2F represents the mean detection *p-values* per sample and shows that all samples mean detection *p-values* were lower than 0.01. To correct for the variability identified in the control probes, a normalization step was applied to the raw intensities using the *preprocessFunnorm* function from *minfi*.

After between-array normalization, different sets of probes that could generate artefacts were removed successively from the methylation dataset: i) 19634 probes on the sex chromosomes, in order to identify differences related to tumors but unrelated to sex chromosomes, ii) 41818 cross-reactive probes which are probes co-hybridizing with multiple CpGs on the genome and not only to the one it has been designed for [34], iii) 10588 probes associated with common SNPs (present in dbSNP build 137), iv) 24363 probes with multi-modal beta-value distribution, and v) 9697 probes having a detection *p*-value higher than 0.01 in at least one sample. Supplementary Table 5 lists the sets of filtered probes. To assess the experimental quality of the assay, the distributions of the beta values were analyzed. As described previously, probes with multi-modal distributions were removed at the filtering step and overall distributions of beta values for each sample before and after filtering were plotted (Figure 2G). As expected, after filtering all samples showed a bimodal profile, indicative of the good quality of the experiment. No experimental batch effects were identified after functional normalization (see Supplementary Fig. 33 from [7]). Based on all the quality controls performed, none of the samples analyzed were identified as outlier. However, one sample available on EGA (201414140007_R06C01), was removed from the analyses because it came from a metastatic tumor rather than the primary tumor. Samples metadata are provided in Supplementary Table 6.

Generation of an integrative molecular map

Here we have generated a pan-LNEN molecular map with the whole-transcriptomic (RNA-Seq) data available from individual studies of each lung NEN tumor type [2, 4, 5, 6, 7, 8]. This dataset includes the RNA-Seq data for a total of 51 SCLC, 69 LCNEC, 118 carcinoids including 40 atypical and 75 typical carcinoids. The different data underwent the same processing steps described above since the generation of the molecular map requires a homogenized dataset.

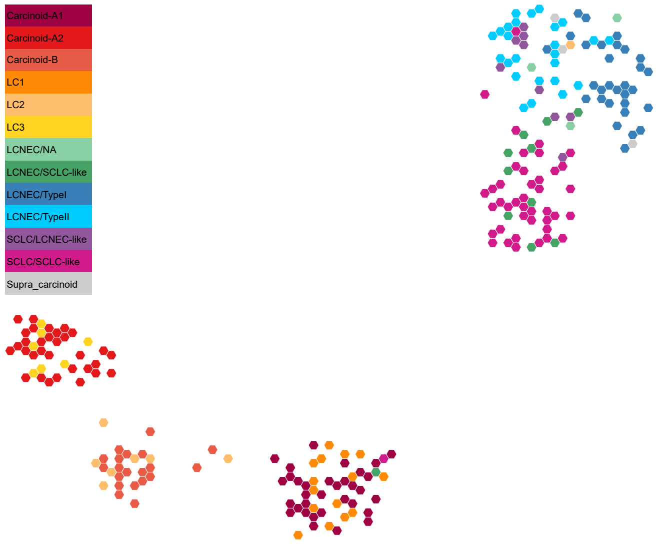

Figure 3. Two dimensional projection of lung NENs transcriptome data using UMAP. The representation was obtained from the TumorMap portal, using the hexagonal grid view, each hexagonal point representing a lung NEN sample. Point colors correspond to the molecular clusters defined in the previous manuscripts.

Dimensionality reduction using UMAP

UMAP method

The pan-LNEN map was obtained using the Uniform Manifold Approximation and Projection (UMAP) method [35] on the genes with the most variable expression (genes explaining 50% of the total variance). UMAP is a dimensionality reduction method based on manifold learning techniques, which are adapted to non-linear data in contrast with the commonly used PCA method. Firstly, it builds a topological representation of the high-dimensional data, and secondly it finds the best low-dimensional representation of this topological structure [35]. UMAP representations were generated using the umap function from the R package umap (v. 0.2.5.0) [36]. All the parameters were set to their default values except the *n_neighbors* parameter. This parameter defines the number of neighbors considered to learn the structure of the topological space. Varying this parameter from small to large values enables the user to find a trade-off between local and global preservation of the space, respectively. In order to preserve the global structure of the data (see "quality control of the UMAP projection" section below), we built the pan-LNEN map setting the *n_neighbors* parameter to 238, which corresponds to the total number of samples.

Biological interpretation of the pan-LNEN TumorMap

Figure 3 shows the pan-LNEN map available on TumorMap [37] (see "Re-use potential" section below), with colors representing the main molecular subtypes. To evaluate the accuracy of the generated pan-LNEN map we firstly verified whether it was consistent with the main biological findings from the original studies, in particular whether it represented the molecular subtypes of lung NENs previously identified, and their relationship with histological types. We specifically tested whether groups of samples previously described as having discordant molecular and histopathological features were identified in our map. To do so, given a focal molecular subtype and two reference histopathological types, we assessed whether samples from the focal molecular subtype were closer to one of the two references using a one-sided Wilcoxon test between the euclidean distances of samples to the centroid of each reference type.

First, the SCLC/LCNEC-like samples [6], which are histological SCLCs presenting the molecular profile of LCNEC, tend to cluster with the LCNECs rather than with the SCLCs (Wilcoxon *p-value* = 6.2×10^{-4}). Similarly, the LCNEC/SCLC-like samples [6], which are histological LCNECs having the molecular profile of SCLC, tend to cluster with the SCLCs rather than with the LCNECs (Wilcoxon *p-value* = 3.3×10^{-3}). In 2018, George *et al.* showed also that LCNEC samples can be subdivided into the type-I and type-II molecular groups [6]. We observed that the type-I and type-II LCNECs were closer to each other than to the SCLC/SCLC-like (Wilcoxon *p-value* = 9.9×10^{-14}) and that SCLC/LCNEC-like samples were closer to type-II than type-I LCNECs [6] (Wilcoxon *p-value* = 3.9×10^{-3}). Like the LCNECs, pulmonary carcinoids have been subdivided in molecular groups. Alcala *et al.* [7] identified three clinically relevant molecular clusters, using a multi-omics factor analysis (MOFA): Carcinoid A1, Carcinoid A2, and Carcinoid B [7]. In the pan-LNEN map generated using UMAP, those three clusters are clearly visible (Figure 3) and respectively correspond to the three clusters identified in [8] named LC1, LC3 and LC2. Also, in the study from Alcala and colleagues [7], two carcinoids that clustered with the carcinoids B (S00118 and S00089) were borderline and located between cluster A1 and B. Similarly, a LCNEC sample and a SCLC sample clustered with the carcinoids A1 [7]. These observations are also visible on the TumorMap representation. Finally, in the same study, a novel entity of carcinoids, named the supra-carcinoids was unveiled. These samples were characterized by a morphology similar to that of pulmonary carcinoids but the molecular features of LCNEC samples. In the pan-LNEN TumorMap, the supra-carcinoids also clustered with the LCNEC samples and were molecularly closer to LCNECs than to SCLCs (Wilcoxon *p-value* = 5×10^{-2}). We also note that one sample from Laddha *et al.* [8] LC2 cluster (SRR7646258) clusters with LCNEC.

Quality control of the UMAP projection

In any dimensional reduction technique, there is a trade-off between preserving the global structure of the data and the fine scale details, and UMAP has been designed to reach a better balance compared to previous methods.

Based on the previously published analyses of lung NEN data [2, 4, 5, 6, 7, 8], we expect the global structure of the data to be composed of six molecular groups (SCLCs, type I and type II LCNECs, Carcinoid A1, A2 and B). For this reason, an ideal projection able to capture this large scale variation should contain five dimensions. To assess the quality

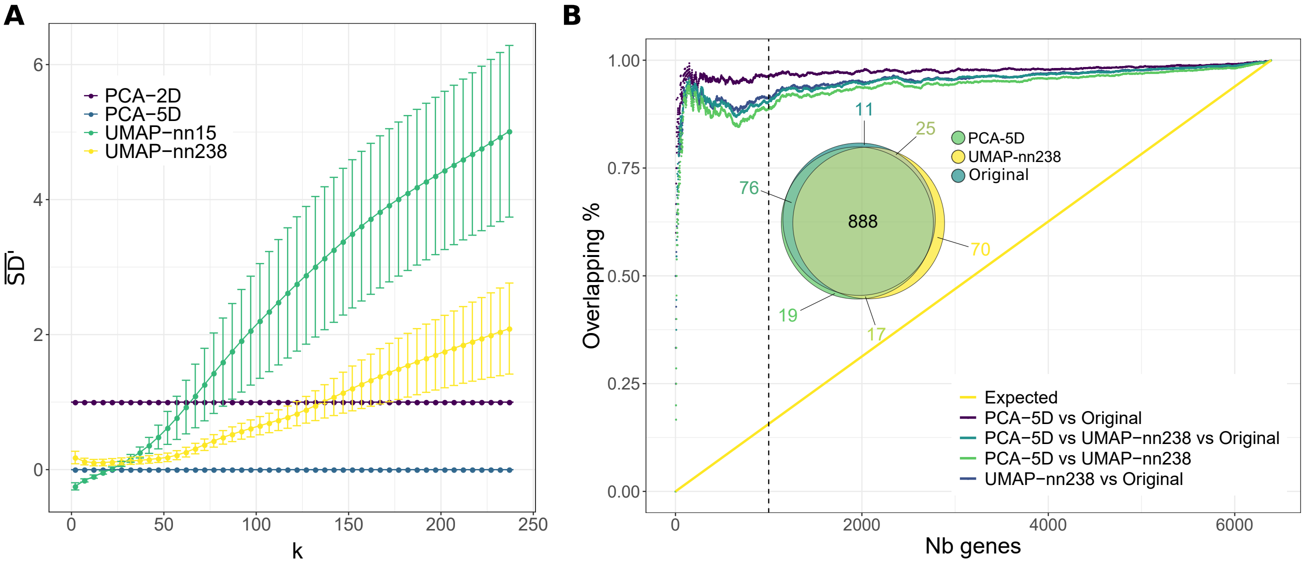

Figure 4. Quality controls performed on the UMAP projection. A) Comparison of the samples' neighborhood preservation for UMAP, PCA-2D, and PCA-5D dimensionality reductions. $\overline{SD}'_k$ values are represented as a function of the number k of nearest neighbors considered, for different dimensionality reduction methods: PCA-2D in purple, PCA-5D in blue, UMAP with $n\_neighbors$ = 238 (UMAP-nn-238) in yellow and UMAP with the default value $n\_neighbors$ = 15 (UMAP-nn-15) in green. Error bars correspond to the means more or less the standard deviations computed across 1000 replicate simulations. **B)** Concordance between gene expressions' spatial auto-correlations in the original space, UMAP-nn-238, and PCA-5D dimensionality reductions. For each space, the genes were ranked based on the spatial auto-correlations of their expression (mean MI values). The concordance is measured as the proportion of overlap between the top N genes in the different spaces (colored lines). The yellow line corresponds to the proportion of overlap expected under the null hypothesis (based on the expected mean of the hypergeometric law). The Euler diagram represents the overlaps between the top 1000 features (N = 1000, dashed line) resulting from the three spaces.

of the 2-dimensional representation generated by UMAP, we propose a comparative analysis between UMAP and the traditional principal component analysis (PCA) based on the five first principal components of PCA (PCA-5D) as implemented in the *dudi.pca* function from the ade4 R package (v1.7-15) [38]. Because UMAP is aiming at preserving the global structure in only two dimensions, we also compared it to the traditional PCA based only on the two first principal components (PCA-2D). We evaluated the performance of the methods based on the preservation of: (i) the samples' neighborhood and (ii) the spatial auto-correlations.

Preservation of the samples' neighborhood

We used the sequence difference view (SD) metric (eq. 3 from [39]) to evaluate the preservation of the samples' neighborhood. This dissimilarity metric compares, for a given sample, its neighborhood in the low-dimensional space with that in the original space, taking into account that preserving the rank of a close neighbor is more important than for a distant neighbor (see [39] for details). SD values are positive ($SD \in [0\,;+\infty)$), with small values indicating a good preservation of the samples neighborhood. We denote by $\overline{SD}_k$ the value of SD averaged across samples for a fixed number of neighbors k; $\overline{SD}_k$ gives a sense of the overall preservation of the neighborhood at different scales: local for low k values and global for large k values. We calculated $\overline{SD}_k$ for PCA-5D, PCA-2D, UMAP with $n\_neighbors$ = 238 and UMAP with the default value $n\_neighbors$ = 15. Because we are interested in the relative values of $\overline{SD}_k$ for the different dimensionality reduction methods, and because we use PCA as a reference, for each dimensionality reduction method X we scaled the values of $\overline{SD}_k$ using that of PCA-5D and PCA-2D:

$$\overline{SD}'_{k,X} = \frac{\overline{SD}_{k,X} - \overline{SD}_{k,PCA-5D}}{\overline{SD}_{k,PCA-2D} - \overline{SD}_{k,PCA-5D}}. \quad (1)$$

By definition, $\overline{SD}'_{k,PCA-5D} = 0$ and $\overline{SD}'_{k,PCA-2D} = 1$. Thus values of $\overline{SD}'_{k,X}$ close to 0 indicate that X preserves k neighborhoods as well as PCA-5D, whereas values close to 1 indicate that X preserves k neighborhoods worse than PCA-5D but as well as PCA-2D, and values greater than 1 indicate that X preserves k neighborhoods worse than PCA-2D and PCA-5D. Note that $\overline{SD}'_{k,X}$ can be negative if X preserves k neighborhoods better than $\overline{SD}_{k,PCA-5D}$. For the UMAP projection, we iterated the computation of $\overline{SD}'_k$ 1000 times, because the algorithm uses a stochastic optimization step to define the projection.

As expected, increasing the $n\_neighbors$ UMAP parameter from 15 to 238 leads to a better preservation of the global structure, clearly visible for $k > 30$ (Figure 4A; mean $\overline{SD}'_{k>30}$ equals to 2.855 and 1.029 respectively), while only marginally reducing the preservation of the local structure for $k < 30$ (mean $\overline{SD}'_{k<30}$ equals to -0.076 and 0.124 respectively), which is approximately the size of the smallest cluster. Globally, the $\overline{SD}'_k$ values over all k levels are lower for a $n\_neighbors$ value of 238 than 15 (paired t-test p-$value$ = 6.09×10^{-8}). With $n\_neighbors$ = 238, the UMAP projection provides a clear improvement over PCA-2D for k around 135 (mean $\overline{SD}'_k < 1$), offering a good trade-off for visualisation in only two dimensions while being able to maintain the global structure of the data, in particular the six molecular groups previously identified. This observation highlights the importance of varying the $n\_neighbors$ parameter according to the purpose of the projection. Some analyses would require to maintain the local structure of the samples neighborhood while others the global structure.

Preservation of spatial auto-correlations

Under the hypothesis that close points on projections share a similar molecular profile, spatial auto-correlations were measured according to the Moran Index (MI) metric [40]. MI values range from -1 to 1, the extreme values indicating negative (nearby locations have dissimilar gene expression) or positive (nearby locations have similar gene expression) spatial auto-correlation, respectively. The spatial auto-correlation of the expression of each gene helps to identify the genes contributing to the structure of the molecular map (MI$\simeq$ 1), and

conversely, the genes that are randomly distributed spatially (MI$\simeq$ 0). The computation of MI requires a weight matrix that determines the spatial scale at which auto-correlation is assessed; we gave a weight of 1 to the k nearest neighbors based on Euclidean distance, and 0 otherwise, so that we can control the scale at which MI is computed with parameter k. The mean MI across k values was computed for all gene expression features for: (i) the original space, (ii) the PCA-5D projection, and (iii) the UMAP projection (with *n_neighbors* = 238). We used the implementation of MI from the Moran.I function of R package ape (v. 5.3) [41].

To evaluate the preservation of the spatial auto-correlations, we ranked the top N genes based on the mean MI values for these three cases and calculated the overlap between the lists (Figure 4B). We found that the PCA-5D is only slightly more conservative of the spatial auto-correlations found in the original space than UMAP (unilateral paired t-test $p.value = 2.2 \times 10^{-16}$). For example, for N = 1000 (see Euler diagram inserted in Figure 4B), 88.8% of the genes with the highest MI overlap between the PCA-5D, UMAP and the original space.

Re-use potential

An interactive TumorMap

Newton and colleagues have recently developed a portal called TumorMap [13, 42], an online tool dedicated to omics data visualization. This new type of integrated genomics portal uses the Google Maps technology designed to facilitate visualization, exploration, and basic statistical interrogation of high dimensional and complex datasets. The pan-LNEN molecular map that we generated in this work (Figure 3) has been shared on the TumorMap platform. Along with the molecular map, the main clinical, histopathological and molecular features highlighted in the previous studies were uploaded as attributes. The interface enables users to explore and navigate through the map: zooming in and out, coloring and filtering samples based on attributes. The users can also create their own attributes based on pre-existing ones by using operators such as union or intersection. In addition, multiple statistical tests are pre-implemented and available, for example: comparison of attributes without considering the samples positions on the map, comparison of attributes considering samples positions on the map, and ordering attributes based on their potential to differentiate two groups of samples. The interactive nature of the map and the fact that its manipulation does not require computational expertise, could enable the generation of new hypotheses and expand the reuse potential of the dataset.

An interactive computational notebook

In the first part of the paper, we described the pre-processing and quality control steps applied on the recently published lung NEN multi-omics dataset [7] in order to facilitate its reuse. To generate the pan-LNEN molecular map, the same pre-processing steps were followed to homogenize independently published transcriptomic data [2, 4, 5, 6, 7, 8]. For that purpose, reproducible pipelines, developed in house, were used and are available for reuse to the scientific community on GitHub [43] (see the "availability of source code" section). In addition, the code used to generate the molecular map and to evaluate the quality of the dimensionality reduction is provided as a notebook published on Nextjournal [44]. Along with the code, the notebook provides the data and the dependencies required to run the analyses performed in this paper. Interested researchers can thus make a copy of this publicly available notebook (called "Remix") to reproduce our results but also interactively modify the code and explore the influence of different parameters.

Integration of new samples

The homogenized read counts of the pan-LNEN data are available on GitHub [14]. Along with the available code, these data could be used to integrate new samples for which RNA-Seq data are available. The raw read counts of the new samples should firstly be generated following the same processing steps described in the section "Data quality controls" (Figure 1, middle panel) and integrated to the pan-LNEN read counts. We also provide in the Nextjournal notebook, the Nextflow command lines allowing to obtain the read counts. The variance stabilized transformation (DESeq2 [32]) should then be applied on the combined data set and UMAP should finally be rerun to project all samples together in a two dimensional space. All together, we provide the resources to integrate additional samples into our molecular map, starting from raw sequencing read counts.

Discussion

Genomic projects focused on rare cancers encounter the limitation of availability of good quality biological material suitable for such studies. This translates in small series of samples usually underpowered to draw meaningful conclusions. Thus, tools facilitating the integration of independent datasets into larger sample series will lead to more informative studies. Recently, the first multi-omic dataset for the understudied atypical pulmonary carcinoids and the first methylation dataset for LCNECs was published [7]. Here we provide a parallel description of the pre-processing of these molecular data and provide evidence of the good quality of the different 'omics data generated. This data collection associated with previous datasets [2, 4, 5, 6, 8] completes the lung NENs molecular landscape and provides thus a valuable resource to improve the molecular characterization of lung NEN tumors. Notably, we show here the perfect concordance of the three molecular clusters of pulmonary carcinoids independently identified in [7] and [8], validating the discoveries made by these two studies and proving the usefulness of this integrative approach.

However, even when primary genomic data is available, barriers to accessing the data still exist, often limiting its reuse by the community [45]. In particular, downloading and re-reprocessing large raw sequencing data requires dedicated infrastructure and bioinformatics skills. Indeed, in order to minimize batch effects when integrating data from different studies, one need to process it exactly in the same way (with the same software and the same versions, the same reference genome, the same annotation databases *etc.*). As more and more data are generated, the previously mentioned reprocessing will become untenable and replicating these efforts for each new study in each research group represents a waste of resources. Standardization of laboratory and computational protocols might become a reality when large national medical genomics initiatives will be fully operational [46]. In the meantime there is a need for better data sharing strategies than the traditional "supplementary spreadsheet / raw data" combination that can accelerate the translational impact of molecular findings.

One step in this direction is the generation of so called "tumor maps", which provide an interactive way to explore the molecular data and allow easy statistical interrogation, includ-

ing generating new hypotheses, but also projecting data from future studies including fewer samples [13]. This integration method has some limitations though. A fixed reference map could be of interest for easier biological interpretations, but the overall sample size of the datasets used to build the pan-LNEN map remains relatively small. Thus, the map does probably not capture the complete molecular diversity of the lung NENs, and integrating new samples will influence the map and potentially change the clusters obtained after dimensionality reduction. Also, if the harmonization of the new dataset to integrate is not enough to correct for strong batch effects, the interpretation of the projections would be erroneous. Another approach would be to project the new samples into a fixed reference map. However, the stochastic nature of UMAP embedding and its sensibility to parameter tuning can lead to unstable projection results, thus this task is for now not straightforward and requires further development [47]. In the meantime, favoring the integration of datasets will, over the years, yield to the constitution of molecular maps that will probably be more and more accurate and more adapted to the projection of new samples.

Conclusion

Here we provide a molecular map based on homogenized transcriptomic data available for the four types of lung NENs from six different studies. We show that this map represents well both the local and global structure of the data, and captures the main biological features previously reported. We provide a full spectrum of data and tools to maximize its re-use potential for a wide range of users: raw sequencing reads, gene expression matrix, bioinformatics pipelines, interactive computational notebooks and an interactive TumorMap. In particular, we indicate how one can update the molecular map by integrating new samples starting from raw sequencing reads. Considering the small sample sizes of molecular studies on rare lung NENs, promoting data integration will empower more reliable statistical testing, and this map will therefore serve as a reference in future studies.

Availability of source code

BAM files have be aligned using the pipeline available at the IARCbioinfo/alignment-nf [48] GitHub repository, revision number 9092214665. The quality control steps applied on our WES and WGS data are available at IARCbioinfo/fastqc-nf [49] and IARCbioinfo/qualimap-nf [50] repositories. The pre-processing steps for methylation arrays are described at the IARCbioinfo/Methylation_analysis_scripts [51] repository. The RNA-Seq analysis steps from the alignment to the raw read counts computation is described at the following GitHub repositories: IARCbioinfo/RNAseq-nf [52] release v2.3, IARCbioinfo/abra-nf [53] release v3.0, IARCbioinfo/BQSR-nf [54] release v1.1 and IARCbioinfo/RNAseq-transcript-nf [55] release v2.1.

Availability of supporting data and materials

The data used in this manuscript are available on the European Genome-phenome Archive (EGA) which is hosted at the EBI and the CRG, under the accession numbers EGAS00001003699, EGAS00001000650, EGAS00001000925, EGAS00001000708, as well as on Gene expression Omnibus (GEO) under GEO SuperSeries GSE118131.

Declarations

Ethical Approval

These data belong to the lungNENomics project, which has been approved by the IARC Ethical Committee.

Consent for publication

Not applicable.

Competing Interests

The authors declare no conflict of interest. Where authors are identified as personnel of the International Agency for Research on Cancer / World Health Organization, the authors alone are responsible for the views expressed in this article and they do not necessarily represent the decisions, policy or views of the International Agency for Research on Cancer / World Health Organization.

Funding

This work has been funded by the US National Institutes of Health (NIH R03CA195253 to L.F.C. and J.D.M.), the French National Cancer Institute (INCa, PRT-K-17-047 to L.F.C. and M.F.), the Ligue Nationale contre le Cancer (LNCC 2016 to L.F.C.), France Genomique (to J.D.M), and the Neuroendocrine Tumor Research Foundation (NETRF, Investigator Award 2019 to L.F.C.). L.M. has a fellowship from the LNCC.

List of abbreviations

AC	Atypical carcinoids
ABRA	Assembly-based realigner
BAM	Binary Alignment Map
CDS	Coding Sequence
CGR	Center for Genomic Regulation
CpG	Cytosine-Phosphate-Guanine
CTAT	The Trinity Cancer Transcriptome Analysis Toolkit
dbSNP	The Single Nucleotide Polymorphism Database
DNA	Deoxyribonucleic acid
EGA	European Genome-phenome Archive
EMBL-EBI	The European Bioinformatics Institute
GATK	Genome Analysis Toolkit
IDAT	File format of the raw methylation data
LCNEC	Large-cell neuroendocrine carcinoma
LCNEC/SCLC-like	Large-cell neuroendocrine carcinomas with the molecular features of small cell lung cancers
LNEN	Lung neuroendocrine neoplasm
MDS	Multidimensional scaling
MI	Moran's Index
MOFA	Multi-omics factor analysis
NEC	Neuroendocrine carcinomas
NEN	Neuroendocrine neoplasm
NET	Neuroendocrine tumors
PCA	Principal Component Analysis
QC	Quality control
RNA-Seq	Ribonucleic acid sequencing
SCLC	Small-cell lung cancer
SCLC/LCNEC-like	Small cell lung cancers with the molecular features of large-cell neuroendocrine carcinomas
SCLC/SCLC-like	Small cell lung cancers with the molecular features of small cell lung cancers
SD	Sequence Difference view metric
SNP	Single Nucleotide Polymorphism
STAR	Spliced Transcripts Alignment to a Reference
TC	Typical carcinoids
TES	Transcription End Site
TSS	Transcription Start Site
UCSC	University of California Santa Cruz
UMAP	Uniform Manifold Approximation and Projection
UTR	Untranslated Transcribed Region
vst	Variance Stabilized Transformation
WES	Whole Exome Sequencing
WGS	Whole Genome Sequencing
WHO	World Health Organization

Additional files

Supplementary Table 1: Samples overview
Supplementary Table 2: Summary table of STAR metrics
Supplementary Table 3: Summary table of RSeQC metrics
Supplementary Table 4: Summary table of HTSeq metrics
Supplementary Table 5: List of filtered probes
Supplementary Table 6: Samples methylation metadata

Author's Contributions

MF and LFC conceived and designed the study. AAGG, EM, NA, LM and CV performed the analyses. VC and AG gave scientific input for the methylation part. JDM helped with logistics and gave scientific input. AAGG, EM, NA, MF and LFC wrote the manuscript. All the authors read and commented the manuscript.

Acknowledgements

This study is part of the lungNENomics project and the Rare Cancers Genomics initiative (www.rarecancersgenomics.com). We also acknowledge the Cologne Centre for Genomics (Cologne, Germany) and the Centre National de Recherche en Génomique Humaine (Evry, France) for generating good quality sequencing data. We also thank Cyrille Cuenin and Zdenko Herceg from the Epigenetics group at IARC; and Teresa Swatloski and Josh Stuart from UCSC for their assistance in hosting our map on the UCSC tumormap portal.

References

1. Rindi G, Klimstra DS, Abedi-Ardekani B, Asa SL, Bosman FT, Brambilla E, et al. A common classification framework for neuroendocrine neoplasms: an International Agency for Research on Cancer (IARC) and World Health Organization (WHO) expert consensus proposal. Modern Pathology 2018;31(12):1770–1786.
2. Peifer M, Fernández-Cuesta L, Sos ML, George J, Seidel D, Kasper LH, et al. Integrative genome analyses identify key somatic driver mutations of small-cell lung cancer. Nature Genetics 2012;44(10):1104–1110.
3. Rudin CM, Durinck S, Stawiski EW, Poirier JT, Modrusan Z, Shames DS, et al. Comprehensive genomic analysis identifies SOX2 as a frequently amplified gene in small-cell lung cancer. Nature Genetics 2012;44(10):1111–1116.
4. Fernandez-Cuesta L, Peifer M, Lu X, Sun R, Ozretić L, Seidal D, et al. Frequent mutations in chromatin-remodelling genes in pulmonary carcinoids. Nature communications 2014;5:3518.
5. George J, Lim JS, Jang SJ, Cun Y, Ozretić L, Kong G, et al. Comprehensive genomic profiles of small cell lung cancer. Nature 2015;524(7563):47–53.
6. George J, Walter V, Peifer M, Alexandrov LB, Seidel D, Leenders F, et al. Integrative genomic profiling of large-cell neuroendocrine carcinomas reveals distinct subtypes of high-grade neuroendocrine lung tumors. Nature Communications 2018;9(1):1048.
7. Alcala N, Leblay N, Gabriel AAG, Mangiante L, Hervas D, Giffon T, et al. Integrative and comparative genomic analyses identify clinically relevant pulmonary carcinoid groups and unveil the supra-carcinoids. Nature Communications 2019;10(1):3407.
8. Laddha SV, Da Silva EM, Robzyk K, Untch BR, Ke H, Rekhtman N, et al. Integrative genomic characterization identifies molecular subtypes of lung carcinoids. Cancer Research 2019;79(17):4339–4347.
9. Pelosi G, Bianchi F, Dama E, Simbolo M, Mafficini A, Sonzogni A, et al. Most high-grade neuroendocrine tumours of the lung are likely to secondarily develop from pre-existing carcinoids: innovative findings skipping the current pathogenesis paradigm. Virchows Archiv 2018;472(4):567–577.
10. Rekhtman N, Pietanza MC, Hellmann MD, Naidoo J, Arora A, Won H, et al. Next-Generation Sequencing of Pulmonary Large Cell Neuroendocrine Carcinoma Reveals Small Cell Carcinoma–like and Non–Small Cell Carcinoma–like Subsets. Clinical Cancer Research 2016;22(14):3618–3629.
11. Simbolo M, Barbi S, Fassan M, Mafficini A, Ali G, Vicentini C, et al. Gene Expression Profiling of Lung Atypical Carcinoids and Large Cell Neuroendocrine Carcinomas Identifies Three Transcriptomic Subtypes with Specific Genomic Alterations. Journal of thoracic oncology : official publication of the International Association for the Study of Lung Cancer 2019;14(9):1651–1661.
12. Fernandez-Cuesta L, Foll M. Molecular studies of lung neuroendocrine neoplasms uncover new concepts and entities. Translational Lung Cancer Research 2019;8(S4).
13. Newton Y, Novak AM, Swatloski T, McColl DC, Chopra S, Graim K, et al. TumorMap: Exploring the Molecular Similarities of Cancer Samples in an Interactive Portal. Cancer Research 2017;77(21):e111–e114.
14. IARCbioinfo/DRMetrics GitHub repository;. https://github.com/IARCbioinfo/DRMetrics, accessed January 2020.
15. Tommaso PD, Floden EW, Magis C, Palumbo E, Notredame C. Nextflow, an efficient tool to improve computation numerical stability in genomic analysis. Biol Aujourdhui 2017;211(3):233–237.
16. Li H, Durbin R. Fast and accurate short read alignment with Burrows-Wheeler transform. Bioinformatics 2009;25(14):1754–1760.
17. Faust GG, Hall IM. SAMBLASTER: fast duplicate marking and structural variant read extraction. Bioinformatics 2014;30(17):2503–5.
18. Tarasov A, Vilella AJ, Cuppen E, Nijman IJ, Prins P. Sambamba: fast processing of NGS alignment formats. Bioinformatics 2015;31(12):2032–2034.
19. Mose LE, Wilkerson MD, Hayes DN, Perou CM, Parker JS. ABRA: improved coding indel detection via assembly-based realignment. Bioinformatics 2014;30(19):2813–5.
20. Andrews S, Krueger F, Segonds-Pichon A, Biggins L, Krueger C, Wingett S, FastQC. Babraham, UK; 2012. http://www.bioinformatics.babraham.ac.uk/projects/fastqc/. Accessed August 2019.
21. Okonechnikov K, Conesa A, Garcia-Alcalde F. Qualimap 2: advanced multi-sample quality control for high-throughput sequencing data. Bioinformatics 2016;32(2):292–294.
22. Ewels P, Magnusson M, Lundin S, Käller M. MultiQC: summarize analysis results for multiple tools and samples in a single report. Bioinformatics 2016;32(19):3047–8.
23. Krueger F, Trim Galore: a wrapper tool around Cutadapt and FastQC to consistently apply quality and adapter trimming to FastQ files, with some extra functionality for MspI-digested RRBS-type (Reduced Representation Bisufite-Seq) libraries; 2012. http://www.bioinformatics.babraham.ac.uk/projects/trim_galore/. Accessed March 2018.
24. CTAT libraries;. https://data.broadinstitute.org/Trinity/CTAT_RESOURCE_LIB/, accessed May 2020.
25. Dobin A, Davis CA, Schlesinger F, Drenkow J, Zaleski C, Jha S, et al. STAR: ultrafast universal RNA-seq aligner. Bioinformatics 2013;29(1):15.

26. Mose LE, Perou CM, Parker JS. Improved indel detection in DNA and RNA via realignment with ABRA2. Bioinformatics 2019 sep;35(17):2966–2973.
27. Depristo MA, Banks E, Poplin R, Garimella KV, Maguire JR, Hartl C, et al. A framework for variation discovery and genotyping using next-generation DNA sequencing data. Nature Genetics 2011 may;43(5):491–501.
28. Van der Auwera GA, Carneiro MO, Hartl C, Poplin R, del Angel G, Levy-Moonshine A, et al. From fastQ data to high-confidence variant calls: The genome analysis toolkit best practices pipeline. Current Protocols in Bioinformatics 2013;11(SUPL.43):11.10.1.
29. Pertea M, Pertea GM, Antonescu CM, Chang TC, Mendell JT, Salzberg SL. StringTie enables improved reconstruction of a transcriptome from RNA-seq reads. Nature Biotechnology 2015;33(3):290–295.
30. Wang L, Wang S, Li W. RSeQC: quality control of RNA-seq experiments. Bioinformatics 2012;28(16):2184–2185.
31. Anders S, Pyl PT, Huber W. HTSeq—a Python framework to work with high-throughput sequencing data. Bioinformatics 2015;31(2):166.
32. Love MI, Huber W, Anders S. Moderated estimation of fold change and dispersion for RNA-seq data with DESeq2. Genome Biology 2014;15(12):550.
33. Aryee MJ, Jaffe AE, Corrada-Bravo H, Ladd-Acosta C, Feinberg AP, Hansen KD, et al. Minfi: a flexible and comprehensive Bioconductor package for the analysis of Infinium DNA methylation microarrays. Bioinformatics 2014;30(10):1363–1369.
34. Pidsley R, Zotenko E, Peters TJ, Lawrence MG, Risbridger GP, Molloy P, et al. Critical evaluation of the Illumina MethylationEPIC BeadChip microarray for whole-genome DNA methylation profiling. Genome Biology 2016;17(1):208.
35. McInnes L, Healy J, Melville J, UMAP: Uniform Manifold Approximation and Projection for Dimension Reduction; 2018. arXiv.
36. Konopka T. umap: Uniform Manifold Approximation and Projection; 2019, https://CRAN.R-project.org/package=umap, r package version 0.2.4.0.
37. pan-LNEN TumorMap;. https://tumormap.ucsc.edu/?p=RCG_lungNENomics/LNEN, accessed July 2019.
38. Dray S, Dufour AB. The ade4 Package: Implementing the Duality Diagram for Ecologists. Journal of Statistical Software 2007;22(4):1–20.
39. Martins RM, Minghim R, Telea AC. Explaining Neighborhood Preservation for Multidimensional Projections. In: Borgo R, Turkay C, editors. Computer Graphics and Visual Computing (CGVC) The Eurographics Association; 2015. .
40. Moran PA. Notes on continuous stochastic phenomena. Biometrika 1950;37(1-2):17–23.
41. Paradis E, Schliep K. ape 5.0: an environment for modern phylogenetics and evolutionary analyses in R. Bioinformatics 2018;35:526–528.
42. TumorMap site;. https://tumormap.ucsc.edu, accessed January 2020.
43. IARC bioinformatics platform;. https://github.com/IARCbioinfo, accessed January 2020.
44. Nextjournal notebook: A molecular map of lung neuroendocrine neoplasms;. https://nextjournal.com/rarecancersgenomics/a-molecular-map-of-lung-neuroendocrine-neoplasms/, accessed January 2020.
45. Learned K, Durbin A, Currie R, Kephart ET, Beale HC, Sanders LM, et al. Barriers to accessing public cancer genomic data. Sci Data 2019 06;6(1):98.
46. Stark Z, Dolman L, Manolio TA, Ozenberger B, Hill SL, Caulfied MJ, et al. Integrating Genomics into Healthcare: A Global Responsibility. Am J Hum Genet 2019 01;104(1):13–20.
47. Espadoto M, Hirata NST, Telea AC. Deep Learning Multidimensional Projections. arXiv 2019;abs/1902.07958.
48. IARCbioinfo/alignment-nf GitHub repository;. https://github.com/IARCbioinfo/alignment-nf, accessed March 2018.
49. IARCbioinfo/fastqc-nf GitHub repository;. https://github.com/IARCbioinfo/fastqc-nf, accessed August 2019.
50. IARCbioinfo/qualimap-nf GitHub repository;. https://github.com/IARCbioinfo/qualimap-nf, accessed August 2019.
51. IARCbioinfo/Methylation_analysis_scripts GitHub repository;. https://github.com/IARCbioinfo/Methylation_analysis_scripts, accessed July 2019.
52. IARCbioinfo/RNAseq-nf GitHub repository;. https://github.com/IARCbioinfo/RNAseq-nf, accessed May 2020.
53. IARCbioinfo/RNAseq-nf GitHub repository;. https://github.com/IARCbioinfo/abra-nf, accessed May 2020.
54. IARCbioinfo/RNAseq-nf GitHub repository;. https://github.com/IARCbioinfo/BQSR-nf, accessed May 2020.
55. IARCbioinfo/RNAseq-nf GitHub repository;. https://github.com/IARCbioinfo/RNAseq-transcript-nf, accessed May 2020.

Supplementary Tables

Click here to access/download
Supplementary Material
Supplementary_tables.xlsx

Track changes manuscript PDF

Click here to access/download
Supplementary Material
versions_comparison.pdf
