# Supplementary material for: A molecular map of lung neuroendocrine neoplasms
Source: Gigascience. 2020 Oct 30;9(11):giaa112. doi: 10.1093/gigascience/giaa112 (PMC7596803; doi:10.1093/gigascience/giaa112)
Supplement: giaa112_Response_to_Reviewer_Comments_Original_Submission [file giaa112_response_to_reviewer_comments_original_submission.pdf]

Author's Response To Reviewer Comments

Close

Dear Editor,

Pleased find enclosed the revised version of our manuscript as well as a a point-by-point response to the reviewers' comments.

We have addressed all the points raised by the reviewers, and importantly, we have integrated the additional dataset proposed by reviewer 2. We are very pleased to see that this new data reinforces our conclusions and thank the reviewer for the suggestion.

Note that we have generated an annotated pdf file to detail and highlight the changes made for this review that can be found as a supplementary file.

Yours sincerely,

Matthieu Foll

Reviewer #1: The authors provide detailed information of multi-omic dataset for a total of 84 lung NENs patients and molecular map integrated with other studies. The high quality multi-omic dataset provided here could help break the study of lung NENs with limited number samples. The detailed process and quality control of generation of an integrative molecular map could help other users to re-use the dataset.
Carrying out open, named peer review I was able to get access to and inspect the data via the IARC Data Access Committee (DAC). Upon applying I got a response from the IARC DAC within a few hours. I was asked to sign a Data Access Agreement before they released the raw data via the EBI EGA. The EGA helpdesk then released the data to my account within 3 days. The data was organized in three datasets according to data type, and users could easily understand what was there. Upon inspection the data stored in the EGA was in accordance with what the authors describe in their accompanying Data Note manuscript.

-> We thank the reviewer for the smooth process during the data access request and signing the required data access agreement.

Minor Revisions:
1. The software used for indel local realignment in manuscript is 'ABRA', the same as the published paper 'Integrative and comparative genomic analyses identify clinically relevant pulmonary carcinoid groups and unveil the supra-carcinoids' , while in pipeline (https://github.com/IARCbioinfo/alignment-nf) is GATK.

-> The option --indel_realignment was not used when running the pipeline alignment-nf on the WES data. Instead, ABRA was run independently afterwards. To clarify this step, we added a sentence in the manuscript page 2 in the paragraph “WES and WGS data‟.

2. A brief introduction of some basic information (e.g. sequencing platform, exome kits) of sequencing data would be helpful.

-> Sequencing information for each omic dataset has been added in the manuscript, page 2 and page 3 in the paragraphs “WES and WGS data” and “RNA-Seq data” respectively.

3. A table with samples (row) and omic data (column) whether present (Yes or No) would be helpful.

-> A supplementary table (Supplementary table 1) has been added, this table contains for each omic a column specifying if the omic data is available or not for each sample. In the text, the table is referenced

page 2 in the section “Data quality controls”.

4. A table with clinical information would be helpful too.
Clinical data such as age, sex, survival and smoking status have been added to the Supplementary Table 1 previously mentioned.

Reviewer #2: The study is well written and present interesting Omics dataset for lung neuroendocrine tumors(LNETs). As these tumors are rare in nature, it is important to create such resources for researcher.
Minor Revisions:
- Using genomics datasets, the authors should able to comment on the tumor purity and provide a table. This is also one important data note for this cancer type.

-> When available, tumor content information has been added to the Supplementary Table 1 previously mentioned.

- Chan CS et al., (PMID:31300474) recently found three novel molecular subtypes of Lung Carcinoids. Authors should consider this dataset and integrate with all lung dataset to enrich for sample size and also one shop center for this cancer type for researcher.

-> We thank the reviewer for pointing us to this interesting additional dataset that we missed. We have now integrated it to our pan-LNEN map and the number of carcinoids considered in the molecular map increased from 88 to 118. Very interestingly this additional dataset allowed us to confirm the existence of three molecular clusters previously identified independently by Alcala et al. and Laddha et al. in 2019. Indeed the clusters LC1, LC2 and LC3 identified by Laddha et al. perfectly matched respectively the cluster A1, B and A2 from Alcala et al. As we had additional data to reprocess, we also took the opportunity to update some software/reference versions at the same time and reprocessed all transcriptomes. Figure 1 middle panel, paragraph “RNA-Seq data” page 3 and 4, Figure 2D and the section “Availability of source code” page 8 have been modified accordingly. Also, the results associated with the molecular map, presented in the section “Generation of an integrative molecular map” have been updated. Finally, the resources complementing this paper, the nextjournal notebook and the DRMetrics GitHub repository have been updated.

Close
