# Supplementary material for: A molecular map of lung neuroendocrine neoplasms
Source: Gigascience. 2020 Oct 30;9(11):giaa112. doi: 10.1093/gigascience/giaa112 (PMC7596803; doi:10.1093/gigascience/giaa112)
Supplement: giaa112_Response_to_Reviewer_Comments_Revision_1 [file giaa112_response_to_reviewer_comments_revision_1.pdf]

Author's Response To Reviewer Comments

Close

Dear Editor,

Following your latest requests (23/09/2020):
- we have added the orcid ID in the pdf directly (I hope this is ok, I couldn't find a way to add the ORCID ID logo and links after each co-author names using the provided Latex template).
- we have added a new reference for the associated GigaDB DOI.
- we have cited this new reference and moved the short source code section text into the Availability of Supporting Data and Materials section.

Pleased find enclosed the revised version of our manuscript. The main changes are:
- we have added the RRIDs of all software cited when available
- we have cited existing tools we used directly in the data description sections rather than in the "Availability of supporting source code and requirements" section
- we have modified accordingly the "Availability of supporting source code and requirements" section

With many thanks for your assistance,

Matthieu Foll

Close
