# Supplementary material for: A molecular map of lung neuroendocrine neoplasms
Source: Gigascience. 2020 Oct 30;9(11):giaa112. doi: 10.1093/gigascience/giaa112 (PMC7596803; doi:10.1093/gigascience/giaa112)
Supplement: giaa112_Reviewer_1_Report_Original_Submission — Fuqiang Li -- 2/25/2020 Reviewed [file giaa112_reviewer_1_report_original_submission.pdf]

Reviewer Report

Title: A molecular map of lung neuroendocrine neoplasms

Version: Original Submission **Date:** 2/25/2020

Reviewer name: Fuqiang Li

Reviewer Comments to Author:

The authors provide detailed information of multi-omic dataset for a total of 84 lung NENs patients and molecular map integrated with other studies. The high quality multi-omic dataset provided here could help break the study of lung NENs with limited number samples. The detailed process and quality control of generation of an integrative molecular map could help other users to re-use the dataset. Carrying out open, named peer review I was able to get access to and inspect the data via the IARC Data Access Committee (DAC). Upon applying I got a response from the IARC DAC within a few hours. I was asked to sign a Data Access Agreement before they released the raw data via the EBI EGA. The EGA helpdesk then released the data to my account within 3 days. The data was organized in three datasets according to data type, and users could easily understand what was there. Upon inspection the data stored in the EGA was in accordance with what the authors describe in their accompanying Data Note manuscript.

Minor Revisions:

1. The software used for indel local realignment in manuscript is 'ABRA', the same as the published paper 'Integrative and comparative genomic analyses identify clinically relevant pulmonary carcinoid groups and unveil the supra-carcinoids' , while in pipeline (https://github.com/IARCbioinfo/alignment-nf) is GATK.
2. A brief introduction of some basic information (e.g. sequencing platform, exome kits) of sequencing data would be helpful.
3. A table with samples (row) and omic data (column) whether present (Yes or No) would be helpful.
4. A table with clinical information would be helpful too.

Level of Interest

Please indicate how interesting you found the manuscript: Choose an item.

Quality of Written English

Please indicate the quality of language in the manuscript: Choose an item.

Declaration of Competing Interests

Please complete a declaration of competing interests, considering the following questions:

- Have you in the past five years received reimbursements, fees, funding, or salary from an organisation that may in any way gain or lose financially from the publication of this manuscript, either now or in the future?
- Do you hold any stocks or shares in an organisation that may in any way gain or lose financially from the publication of this manuscript, either now or in the future?
- Do you hold or are you currently applying for any patents relating to the content of the manuscript?
- Have you received reimbursements, fees, funding, or salary from an organization that holds or has applied for patents relating to the content of the manuscript?
- Do you have any other financial competing interests?
- Do you have any non-financial competing interests in relation to this paper?

If you can answer no to all of the above, write 'I declare that I have no competing interests' below. If your reply is yes to any, please give details below.

I declare that I have no competing interests.

I agree to the open peer review policy of the journal. I understand that my name will be included on my report to the authors and, if the manuscript is accepted for publication, my named report including any attachments I upload will be posted on the website along with the authors' responses. I agree for my report to be made available under an Open Access Creative Commons CC-BY license (http://creativecommons.org/licenses/by/4.0/). I understand that any comments which I do not wish to be included in my named report can be included as confidential comments to the editors, which will not be published.

Choose an item.

To further support our reviewers, we have joined with Publons, where you can gain additional credit to further highlight your hard work (see: https://publons.com/journal/530/gigascience). On publication of this paper, your review will be automatically added to Publons, you can then choose whether or not to claim your Publons credit. I understand this statement.

Yes Choose an item.
